# Fermented Fish Products: A Comprehensive Overview of Traditional Processing Techniques, Varieties, and Their Health Benefits

**DOI:** 10.1111/1541-4337.70457

**Published:** 2026-04-21

**Authors:** Banlambhabok Khongthaw, Mthokozisi Dladla, Pankaj Kumar Chauhan, Kanika Dulta, Vinod Kumar, Helen Oneyaka, Soumya Ghosh

**Affiliations:** ^1^ School of Bio‐engineering and Food Technology Faculty of Applied Sciences and Biotechnology, Shoolini University Solan Himachal Pradesh India; ^2^ Human Molecular Biology Unit (School of Biomedical Sciences), Faculty of Health Sciences University of the Free State Bloemfontein South Africa; ^3^ Center of Advanced Innovation Technologies VŠB‐Technical University of Ostrava Ostrava‐Poruba Czech Republic; ^4^ Department of Chemistry and Biochemistry Mendel University in Brno Brno Czech Republic; ^5^ Algal Research and Bioenergy Laboratory, Department of Food Science and Technology Graphic Era (Deemed to be University) Dehradun Uttarakhand India; ^6^ Peoples' Friendship University of Russia (RUDN University) Moscow Russian Federation; ^7^ School of Chemical Engineering University of Birmingham Edgbaston Birmingham UK; ^8^ Natural and Medical Sciences Research Center University of Nizwa Nizwa Oman; ^9^ School of Bio‐engineering and Food Technology University of the Free State Bloemfontein South Africa

**Keywords:** bioactive peptides, fermented fish, health benefits, lactic acid bacteria, probiotics

## Abstract

This review addresses the processing aspects and integrates in vitro and in vivo findings to highlight the various health benefits of indigenous fermented fish products from different regions of the world. Across Asia, Africa, and Northern Europe, fermented fish holds cultural significance, with each region boasting unique varieties shaped by local traditions. This literature examined published articles on the various therapeutic properties of fermented fish by focusing on its lactic acid bacteria (LAB), antioxidant, anticancer, gut microbiota, and antimutagenic activities. Fermentation of fish products using starter cultures of *Lactobacillus*, *Enterococcus*, and *Monascus purpureus* enhanced the production of peptides and improved digestibility. Fermented fish substrates inoculated with *Lactobacillus* and *Bacillus* spp. generated bioactive peptides (e.g., FSGLR, IAEVFLITDPK, NVPVYEGY) with antioxidant and antihypertensive potential, thereby enhancing the nutritional and functional quality of traditional fermented fish products. These peptides modulated gut microbiota (↑Bacteroidetes, ↓Firmicutes) and metabolites, while fermented fish collagen suppressed oxidative stress (↓NOX, ↓MITF, ↓TYR) and activated GlyR/GlyT and SOD pathways. Fermented fish products are valuable sources of antioxidant and anticancer peptides. However, further research is needed to improve food safety by enhancing hygiene practices and controlling microbial contaminants.

**Abbreviations**
ACE: angiotensin‐converting enzyme
AIPPHPYP: Ala‐Ile‐Pro‐ProHis‐Pro‐Tyr‐Pro
Ala: alanine
Arg: arginine
Asp: aspartic acid
BPs: bioactive peptides
CLA: conjugated linoleic acid
Cys: cysteine
DAGPYGPI: Asp‐Ala‐Gly‐Pro‐Tyr‐Gly‐Pro‐Ile
DNA: deoxyribonucleic acid
DOCA: deoxycorticosterone acetate
EMGPA: Glu‐Met‐ Gly‐Pro‐Ala
ERGPLGPH: Glu‐Arg‐Gly‐Pro‐Leu‐Gly‐Pro‐His
FFA: free fatty acids
FPH: fish protein hydrolysates
GABA: γ‐aminobutyric acid
GC/IMS: gas chromatography/ion mobility spectrometry
GC/MS: gas chromatography/mass spectrometry
GI: gastrointestinal
Glu: glutamic acid
HACCP: analysis and critical control points
HepG2: hepatocellular carcinoma cell
HFP: histamine fish poisoning
HIV: human immunodeficiency virus
HPLC: high‐performance liquid chromatography
HS–SPME–GC–MS: solid‐phase microextraction–gas chromatography–mass spectrometry
IAEVFLITDPK: Ile‐Ala‐Glu‐Val‐Phe‐Leu‐Ile‐Tre‐Asp‐Pro‐Lys
IFRPD P15 strain: Institute of Food Research and Product Development P15 strain
Ile: isoleucine
KNOS: kinin–nitric oxide systems
LAB: lactic acid bacteria
Lys: lysine
Met: methionine
MSG: monosodium glutamate
Phe: phenylalanine
PO: prolyl oligopeptidase
RAS: renin–angiotensin
SPME–GC–MS: solid‐phase microextraction–gas chromatography–mass spectrometry
TCA: trichloroacetic acid
TMA: transformed trimethylamine
UHPLC‐QE‐MS/MS: ultrahigh‐performance liquid chromatography coupled with quadrupole‐orbitrap mass spectrometry/mass spectrometry
Val: valine
VEE: Val‐Glu‐Glu
WMFDW: Trp‐Met‐Phe‐Asp‐Trp
WMGPY: Trp‐Met‐Gly‐Pro‐Tyr

## 1 Introduction

Fermentation is a traditional method used to preserve and enhance food. It is an effective food processing and preservation technique, widely applied to vegetables, fish, and meat, offering benefits such as enriched flavor and extended shelf life. Fermented fish products are rich in minerals, proteins, selenium, omega‐3 fatty acids, and vitamin D, making them an essential part of a balanced diet (Ghosh et al. 2024; Han et al. 2023). Fermented fish, integral to many global food traditions such as southeast Asian thai pal, and sushi, are the major industry and a source of valuable microbes. Despite the presence of live microorganisms (probiotics) in fermented food, the health advantages of consuming them remain uncertain. While probiotics may support gut health, their benefits vary by bacterial strain, individual health, and dosage, requiring more research to fully understand their effects. It is consumed worldwide, reflecting diverse culinary cultures (Siddiqui et al. 2023). Fermentation is a cost‐effective and well‐established method of food preservation. The traditional technology was researched through discussions with local producers, revealing its roots and its conversion from drying to fermenting fish, as described by the community's elders (Majumdar et al. 2016a; Svanberg 2023).

On the other hand, fish is globally acclaimed as a super‐food, with an average consumption of 20.2 kg per person annually. Aquatic systems provide 157.4 million tons of food rich in easily digestible proteins, essential amino acids, and health‐beneficial peptides. India contributes 8% (162.48 lakh tones) to global fish production, but per capita consumption is below the global average at 6.31 kg. Both global and Indian fish production suffer significant losses of 27%–39% due to postharvest and process‐related waste (Ravishankar and Elavarasan 2024). Fish have high water activity, neutral pH, and autolytic enzymes, making them prone to rapid microbial growth, oxidative degradation, and spoilage. Microbial enzymes degrade proteins and other organic ingredients into smaller compounds like amines, peptides, and nitrogenous substances, which contribute to objectionable aroma synthesis (Zhang et al. 2023). On the other hand, fermentation methods extend their shelf life, enhance their quality and aroma (Feng et al. 2021; Ndudi et al. 2024).

Fish fermentation is an ancient process, with evidence showing it was commonly used in Japan during the Yayoi period (300 BCE to 300 CE; Chan et al. 2023). Throughout history, there exit cultures to preserve fish by drying, salting, and fermenting to ensure food security during periods of scarcity after abundant seasonal fish supplies (Narzary et al. 2021). Increased fish production results in more waste, emphasizing the need to preserve this valuable nutrient source. Fermentation extends the short shelf life of fresh fish through a metabolic process that derives energy from organic compounds without external oxidizing agents. Additionally, fermented fish products can significantly boost the national economy. For instance, Thailand's leadership in the production and export of fermented fish sauce greatly contributes to its economic growth (Chan et al. 2023). Fermented fish comes in various shapes, sizes, and preparations, including fish sauce, paste, and solid dried forms such as tungtap from Meghalaya, India. Compared to smoking, freezing, and drying, fermentation is a superior preservation method for fish, enhancing its nutritional value through the growth of beneficial microorganisms such as *Bifidobacterium*, *Leuconostoc*, *Lactobacillus*, *Streptococcus enterococcus*, and *Lactococcus* (Canon et al. 2020; Ray et al. 2023). Fermented fish differs worldwide due to cultural, social, and environmental influences. Notable examples include lonailish, godak from India, rakfisk from Norway, nampla from Thailand, and patis from the Philippines. These products can be categorized into whole fish bodies, surimi, fish sauce, and fish soy sauce (Martí‑Quijal et al. 2020; Narzary et al. 2021; Tamang et al. 2020). Recent research on probiotics from Malaysian fermented fish product, Pekasam, identified three strains with potent antimicrobial activity against *Escherichia coli*, *Staphylococcus aureus*, and *Klebsiella* spp. These strains show promising potential as biotherapeutic agents (Ida Muryany et al. 2017). This review aims to comprehensively examine indigenous fermented fish products from different regions of the world by integrating traditional processing techniques with recent scientific evidence on their nutritional, functional, and health‐promoting properties. Beyond cataloguing regional varieties, the review highlights the pivotal role of lactic acid bacteria (LAB) and other functional microorganisms in driving biochemical transformations that lead to the formation of bioactive peptides (BPs), antioxidant compounds, and gut microbiota–modulating metabolites. A distinctive feature of this work is the synthesis of in vitro and in vivo findings that elucidate the mechanistic basis of the reported therapeutic effects, including antihypertensive, antioxidant, anticancer, antimutagenic, and antimicrobial activities. In addition, the review critically discusses safety concerns, microbial and chemical risks, and processing challenges associated with traditional fermentation practices. By linking cultural knowledge with advances in fermentation science, microbial ecology, and food safety, this review identifies key research gaps and future directions needed to optimize product quality, enhance health benefits, and support the sustainable development of fermented fish products for broader food and public health applications.

## 2 Classification of Indigenous Technology Processes

**FIGURE 1:**
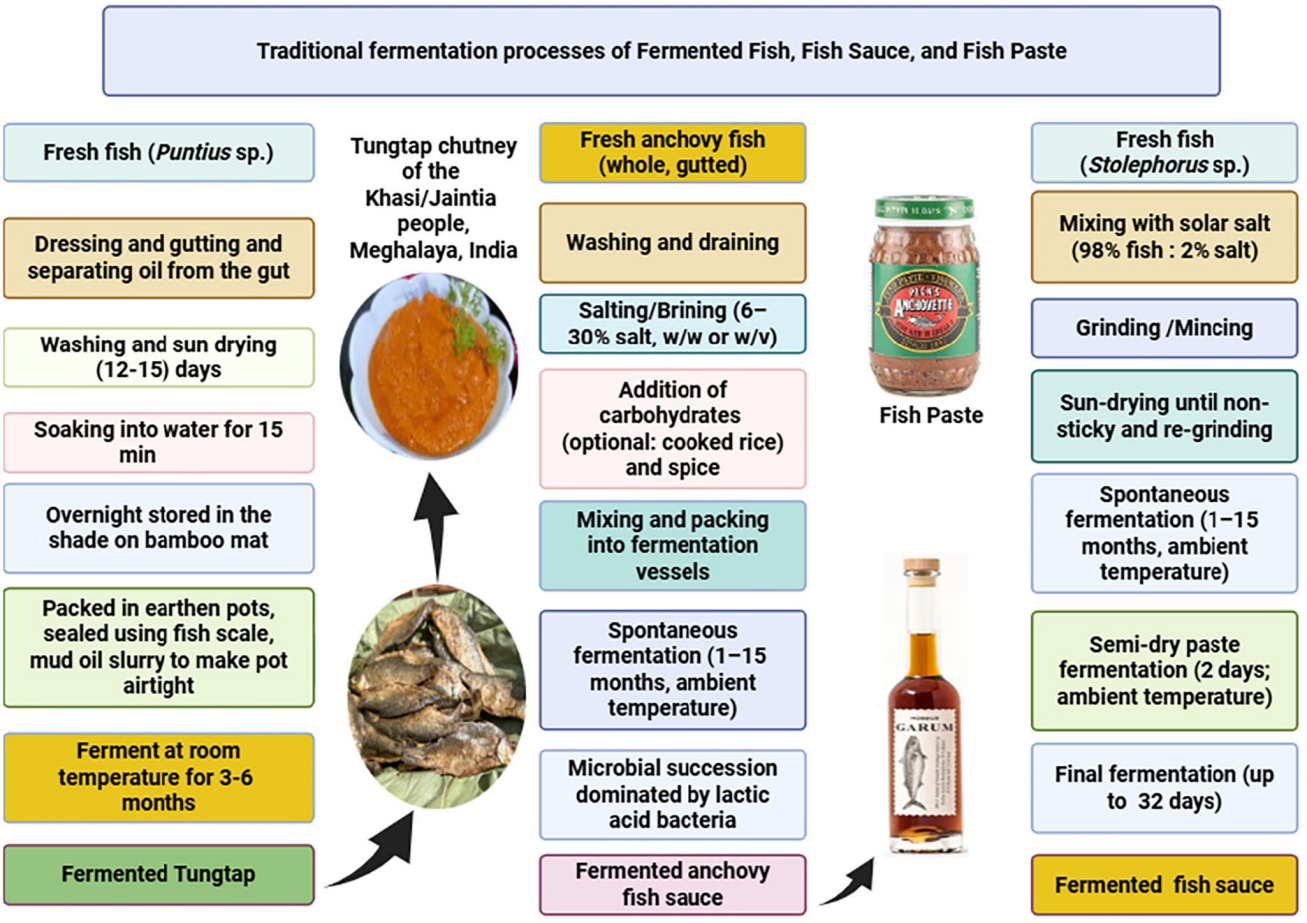
Flow diagram of traditional production processes of three types of fermented fish, fish sauce, and fish paste.

Codifying fermented fish products involves several criteria: processing technique, substrates utilized, salt content, and the physical characteristics of the final product. The traditional fermentation process is uncontrolled, and consequently, there is no standard for the production. The raw materials used for processing include fish and salt, and the primary limitations observed by processors are fermentation time and storage conditions (Nampoothiri et al. 2017; Sharma et al. 2022). Traditional fermented fish products fall into three main categories: (a) those fermented without carbohydrates, relying on bacterial enzymes from the fish–salt mixture; (b) those with carbohydrates in a fish–carbohydrate mixture; and (c) those using a starter culture to ferment carbohydrates (Nath et al. 2024). Primary forms include (a) original form products (e.g., buro, pedah‐kemburg, Makassar, nigari, and Colombo cured mackerel), (b) liquid products (e.g., budu, nampla, patis, and nuoc‐mam), and (c) pastes (e.g., prahok, trasi, ngapi, bagoong, and belacan; Narzary et al. 2021; Figure 1).

**FIGURE 2:**
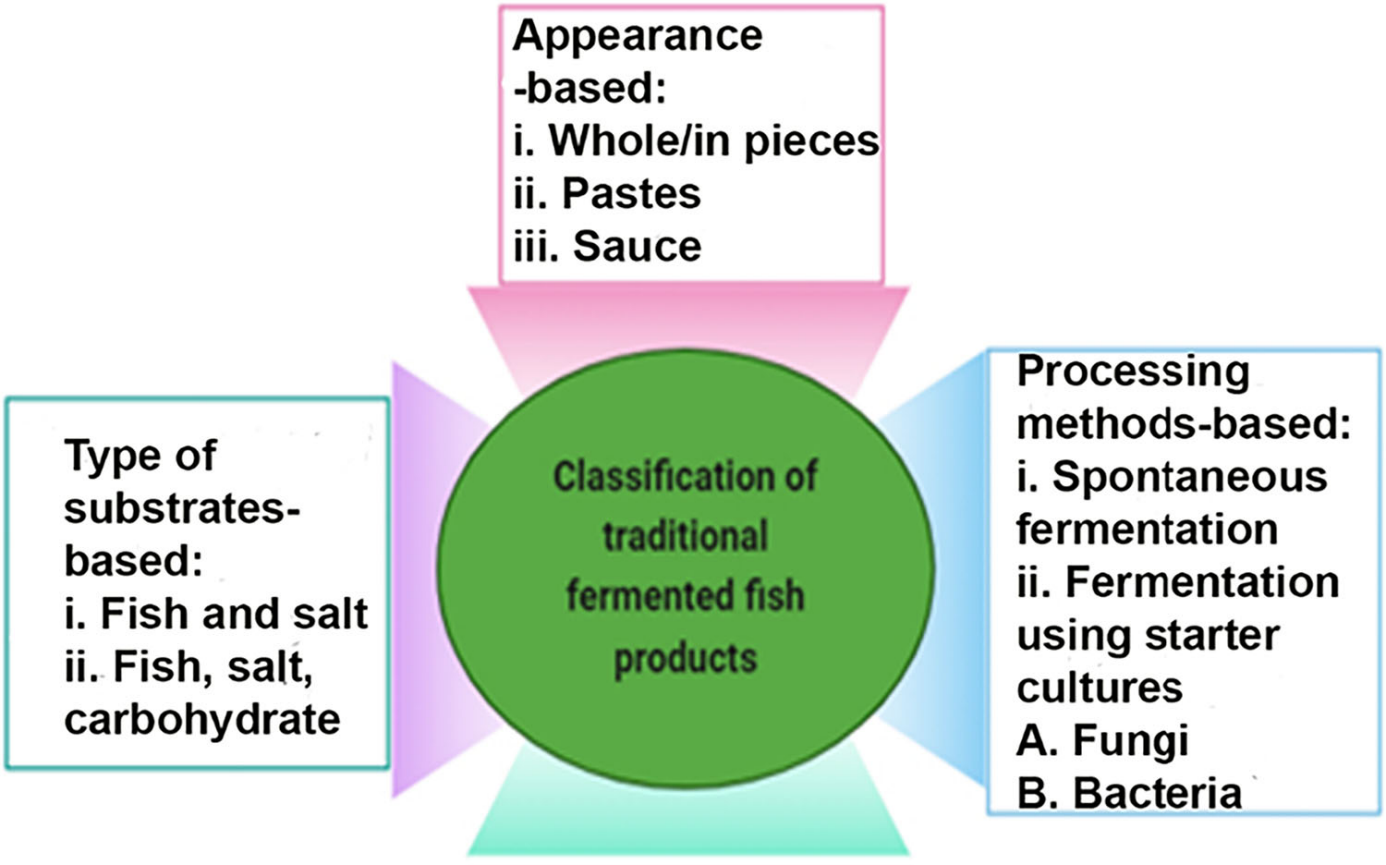
Classification of traditional fermented fish products.

In spontaneous fermentations, mixed cultures from raw materials are common, while industrial processes favor standardized starter cultures for consistent quality, though these can lead to reduced biodiversity over time (Russo et al. 2017). Traditional fermentations leverage robust LAB strains that produce antimicrobial compounds like bacteriocins, enhancing safety, texture, flavor, and probiotic qualities. Gene technology advancements have allowed for starter cultures tailored with specific traits (Anumudu et al. 2024). Starter cultures, comprising single or mixed strains, initiate and accelerate fermentation, with LAB providing controlled, predictable outcomes (Taskila 2017). Fungal fermentation reduces pathogens and enhances flavor, as mold starters enrich nutrition and taste in fish pastes (Sionek et al. 2023). Mixed cultures of *Halobacterium* and *Clostridium* spp. increase amino acids such as glutamate, alanine, and leucine, and nonvolatile compounds such as aldehydes, alcohols, ketones, and esters in golden pomfret fermentation (Qiu et al. 2022). Similarly, mixed starter fermentation with *Enterococcus lactis* and *E. rivorum*, along with natural fermentation, enhances protease activity, trichloroacetic acid (TCA) soluble protein levels, and flavor components in suanyu fermentation. Volatile aldehydes such as caprylic, trans‐2‐nonenal, and pelargonic influencing a significant role in shaping the product's flavor profile, as indicated by relative odor activity levels (Yuan et al. 2023). The inoculation of *Halanaerobium fermentans* YL9‐2 from fermented fish sauce significantly increases amino acid nitrogen, reduces biogenic amines, and improves product quality and aroma (Wenjing et al. 2022). The inoculation of fermented fish sauce with *Virgibacillus* sp. or *T*. *halophilus* strains either sequentially or as a single culture, enhanced the fishy flavor and umami (Udomsil et al. 2017). The mixed starter cultures used for suanyu fermentation, including *Staphylococcus*, *Macrococcus*, and *Lactobacillus*, along with spices, enhance the quality and safety of the product by inhibiting undesirable microorganisms such as *Enterococcus*. The activity of these cultures plays a key role in acidification and stimulates the development of volatile compounds (Sun et al. 2022). The inoculation of commercial starter strains, such as *Staphylococcus xylosus* and *Pediococcus lactis*, improved microbial quality by inhibiting pathogenic microorganisms, and contributes to higher free amino acid (FAA) formation, and enhanced flavor in fermented fish products (Hua et al. 2020). This classification is illustrated in Figure 2.

## 3 Lactic Acid Bacteria Fermented Fish

The concept of LAB was introduced in the early 1900s, with the first pure culture, *Bacterium lactis* (now *Lactococcus lactis*), isolated by Lister in 1873 (Liu et al. 2012). Orla‐Jensen coined the term “Lactic acid bacteria” in 1919, forming the foundation for today's classification system (Vinderola et al. 2019). LAB plays critical roles in food fermentation, pharmaceuticals, and specialized diets, producing lactic acid as a major end product from glucose, which supports energy production via deaminase and decarboxylase enzymes. Common LAB strains in food include *Lactobacillus*, *Streptococcus*, *Lactococcus*, *Aerococcus*, *Enterococcus*, *Leuconostoc*, *Pediococcus*, and *Bifidobacterium*, along with bacilli like *Bifidobacteria* and *Actinomyces Israeli*, which also generate lactic acid (Al‐Maqtari et al. 2019; Belleggia and Osimani 2023). The LAB isolated from the traditional fermented fish shidal were *Lactiplantibacillus plantarum*, *E*. *lactis*, *E*. *faecalis*, *Enterococcus faecium*, *P. pentosaceus*, and *Pediococcus acidilactici* (Gupta et al. 2021), whereas *L. buchneri*, *S. harbinensis*, *L. parabuchneri*, *L. harbinensis*, and *L*. *parabuchneri* were isolated from fermented fish badu (Ardani et al. 2023). The probiotics isolated from the fermented fish dish pla‐paeng‐daeng include *Lactiplantibacillus pentosus*, *Lactiplantibacillus argentoratensis*, *Limosilactobacillus fermentum*, *Lactobacillus companii*, *Lactobacillus farciminis*, *Lactobacillus futsaii*, and *E. lactis* (Kingkaew et al. 2023). During fermentation, LAB converts lactose to lactic acid, releasing BPs that improve protein digestibility, release fatty acids, and synthesize bioactive compounds used in pharmaceuticals and nutraceuticals (Sharma et al. 2023). Biopreservation employs natural microbiota and antimicrobials to prolong food shelf life. LAB and its products, including bacteriocins like nisin, prevent spoilage and combat pathogens with their antimicrobial properties. These bacteriocins effectively preserve food by inhibiting foodborne pathogens such as *S. aureus, E. coli*, and *L. monocytogenes* (Singh 2018). In some of the fermented fish and fermented food products where there is a sufficient amount of carbohydrates, LAB utilizes a sophisticated proteolytic system to break down a substance (peptide and protein) into oligopeptides and FAAs (Wu et al. 2025). LAB are integral in various fermented foods, including yogurt, cheese, kimchi, sauerkraut, salami, sourdough, and fish products like tungtap, suanyu, and shidal. *Lactobacillus* strains, such as *P. acidilactici*, are robust probiotics with antioxidant, antibiotic resistance, and enzyme production capabilities, making them ideal for food and feed applications. Consuming LAB‐fermented fish enhances nutritional value, introduces beneficial compounds like bacteriocins, and inhibits harmful bacteria, reducing biogenic amines in the body (Ray et al. 2024). *L. plantarum*, a promising probiotic, exhibits anti‐inflammatory, antimicrobial, antioxidant, antigenotoxic, and immunomodulatory effects and has broad antibacterial activity against spoilage microbes and pathogens, including *Salmonella*, L*. monocytogenes*, *S. aureus*, and *Bacillus* spp., and *E. coli* (Coulibaly et al. 2023).

Consuming fermented fish products containing LAB enhances nutritional value and produces compounds like bacteriocins. These antimicrobials substances inhibit the growth of pathogenic microorganisms and help reduce the accumulation of biogenic amines in the body (Hernández‐González et al. 2021; Vieco‐Saiz et al. 2019). Multiple research studies have highlighted *L. plantarum* as a promising probiotic strain for applications in pharmaceutical and food industries. These LAB strains offer health benefits, including anti‐inflammatory, antimicrobial, antioxidant, antigenotoxic, and immunomodulatory effects (Garcia‐Gonzalez et al. 2021). *L*. *plantarum* also has broad antibacterial activity against many food spoilage microbes and pathogens, including *E*. *coli*, *Bacillus* spp.*, L*. *monocytogenes*, *Clostridium*, *P*. *aeruginosa*, *H*. *pylori*, *Klebsiella* spp., *Salmonella*, *S*. *aureus*, *Enterococcus*, and *Shigella* (Dinev et al. 2018; Hernández‐González et al. 2021). The production of bacteriocins by *L*. *plantarum* exhibited antimicrobial activity against foodborne spoilage and pathogenic bacteria. It was reported that it is effective against *Listeria monocytogenes* 54002, *S. aureus* 13565, *Lactobacillus delbrueckii* subsp. lactis, *L. plantarum* S‐35, *Lactobacillus bulgaricus*, and *Lactococcus lactis* NZ9000, with inhibition zones measuring 16.4 ± 0.23, 9.12 ± 0.42, 18.81 ± 0.12, 17.32 ± 0.33, 14.32 ± 0.29, and 14.43 ± 0.21 mm, respectively (Wang et al. 2018). *L*. *plantarum* and *E*. *faecium* have been identified as the dominant strains utilized as starter cultures in mahyaveh fermentation. These starter cultures enhance the flavor, texture, and nutritional quality and increase the shelf‐life of fermented foods. *Lactobacillus sakei* subsp. sakei was isolated from stinky mandarin fish using traditional microbial culture techniques. The bacteriocin sakacin C2 produced by this strain exhibited strong antimicrobial potential against pathogenic microorganisms (gram‐positive and gram‐negative), notably inhibiting the growth of *E*. *coli*, *S*. *aureus*, *B*. *subtilis*, and *P*. *aeruginosa* with inhibition zones of 11.27 ± 0.21, 11.70 ± 0.44, 12.03 ± 0.35, and 10.63 ± 0.12 mm, respectively (Karparvar et al. 2019). *Lactobacillus sakei* subsp. strains, isolated from fermented mandarin fish, demonstrated effective inhibition of foodborne spoilage. They exhibited inhibition zones of *B*. *subtilis* (12.03 ± 0.35 mm), *S*. *aureus* (11.70 ± 0.44 mm), *E*. *coli* (11.27 ± 0.21 mm), and *P*. *aeruginosa* (10.63 ± 0.12 mm), respectively (Wu et al. 2022). *Tetragenococcus* bacteria, known as halophilic bacteria, are frequently present in high salt fermented fish and other fermented foods. Studies have shown that exopolysaccharide fractions produced by *Tetragenococcus* strains possess antioxidant activity, cryoprotective properties, and moderate emulsifying capacity, indicating their promising applications as starters in the fermentation food industry (Belleggia and Osimani 2023).

## 4 Role of Lactic Acid Bacteria in the Fermented Fish Aroma

LAB significantly contributes to the aroma profile of fermented fish products by producing nonvolatile (e.g., lactic, malic, acetic, citric, and tartaric acids) and volatile compounds (e.g., phenolic, ethanol, ethyl ether, aldehydes, phenols, ketones, hexanal, and heptanal) through metabolic reactions such as hydrolysis, proteolysis, and lipolysis (Simon 2021; Table 1). The distinctive aroma of fermented fish serves as an important sensory indicator of the physical and chemical transformations in the raw substrate. Free fatty acids (FFAs) and FAAs, resulting from lipolysis and proteolysis by LAB, respectively, are critical precursors to volatile aroma compounds. Lipid oxidation further influences aroma perception, stability, and production (Dei 2012; Ziarno and Cichońska 2021). Fermented fish aromas vary widely, from sweet and salty to sour, bitter, and spicy, influenced by preparation methods, sources, and ingredients. Sourness and umami are particularly significant, with sourness primarily arising from organic acids, especially lactic acid, and other acids (e.g., propionic, n‐butyric, isobutyric, n‐valeric formic, and isovaleric) contributing to the aroma (Cai et al. 2024; Q. Guo et al. 2023). The introduction of LAB accelerates lactic acid production, indicating homolactic fermentation, while stable acetic acid levels suggest its secondary role in aroma development (An et al. 2022).

**TABLE 1:** Lactic acid bacteria effects on the aroma of fermented fish products.

Sample	Isolated LAB	Analysis	Nonvolatile compound	Volatile compound	Aroma	References
*Cyprinuscarpio* L. (Suanyu)	*L. plantarum*; *P*. *pentosaceus*; *S*. *cerevisiae*	GC/MS	ND	↑Aldehydes; ↑acids; ↑alcohols; ↑esters; ↑ketones	Unique wine flavor; moderate acid taste	Zeng et al. (2017)
Suanyu	*Lactiplantibacillus plantarum*; *S*. *cerevisiae*	GC/MS	↑Asp; ↑Glu; ↑Lys; ↑Ile; ↑Cys; ↑Ala; ↑Phe	↑Alcohols; ↑aldehydes; ↑ketones; ↑esters; ↑aromatic compounds; ↑acids; ↑hydrocarbons	Umami; sweet amino acids	Zhang et al. (2022)
*Siniperca chuatsi* (mandarin fish)	*Pediococcus pentosaceus*; *P*. *lactis*; *L*. *plantarum*; *L*.* sakei*	UHPLC‐QE‐MS/MS	↑Asp; ↑Glu	—	Sweet; umami; bitter amino acids	Wang et al. (2022)
Chouguiyu	*Fusobacterium*; *Arcobacter*; *Oceanisphaera*	GC/MS	↑Lle; ↑Ser; ↑Pro; ↑Leu; ↑Tyr; ↑Phe; ↑His; ↑Arg; ↑Glu; ↑Gly; ↑Ala; ↑Val; ↑Met	↑Alcohols; ↑ketones; ↑nitrogenous; ↑compounds; ↑hydrocarbon	Blend of floral; minty; sweet; slightly pungent	Yang et al. (2020)
Golden pomfret (*Trachinotus ovatus*)	*Halobacterium*; *Clostridium*; *Natrinema*; *Alkalibacillus*; *Natrialba*; *Vibrio*	GC/IMS; HPLC; amino acid analyzer	↑Glu; ↑Ala; ↑Leu	↑aldehydes; ↑alcohols; ↑ketones; ↑esters	Savory; umami	Qiu et al. (2022)
*Pneumatophorus japonicus* (mackerel)	*Lactiplantibacillus plantarum* LP1; *Weissella cibaria* WC1	GC/MS	↑Glu; ↑Ala; ↑Ser; ↑Gly; ↑Thr; ↑Ala	↑aldehydes; ↑alcohols	Strong umami; pleasant savory	Zhou et al. (2021)
Carp	*Enterococcus*; *Lactobacillus*; *Lactococcus*; *Leuconostoc*; *Staphylococcus*	SPME–GC–MS	↑Glu; ↑Ser; ↑Pro; ↑Gly; ↑Ala; ↑Val; ↑Tyr; ↑Asp; ↑Phe; ↑His; ↑Arg	—	Sour; tangy; slightly cheesy	Wang et al. (2021)
Pla‐ra	*Micrococcus *spp.;* Bacillus subtilis*; *Pediococcus halophilus*	GCMS	Glu; Ala; Leu	Aldehyde; alcohol; ester; furan; pyrazine; sulfur; ketone	Savory; sweet; roasted; slightly pungent; umami	Loyda et al. (2023)
*Surimi tilapia*	*Latilactobacillus sakei*; *Pediococcus acidilactici*	HS‐SPME–GC–MS	—	Alcohols; ketones; aldehydes	Fishy; sulfurous odors	Li et al. (2024)
Suanyu (*Carassius auratus*)	*Lactococcus enterotis*;* Enterococcus rivorum*	GC/MS	—	↑Aldehydes (octanal, nonanal, (E)‐2‐nonenal)	Umami	Yuan et al. (2023)

Specific LAB strains identified in *Chouguiyu*, a traditional Chinese fermented fish, including species such as *Enterococcus*, *Peptostreptococcus*, *Acinetobacter*, *Psychrobacter*, and *Vagococcus*, contribute significantly to the development of umami peptides. *Chouguiyu* also contains volatile compounds like indole, trimethylamine sulfur compounds, esters, phenols, acetic acid, and aldehydes (Yang et al. 2022). LAB such as *Lactobacillus* spp., *Staphylococcus* spp., *Megacoccus* spp., *Wickerhamomyces anomalus*, and *Candida* spp. contribute to the distinctive flavors of sour fish both pre‐ and postfermentation (Mao et al. 2024). Fermented *Siniperca chuatsi* contains compounds like linalool, trimethylamine, indole, and geranyl acetate, shaped by LAB strains such as *Psychrilyobacter*, *Fusobacterium*, and *Acidaminococcus* (Chen et al. 2023). In Zhuyu, another fermented fish, strong umami flavors result from the starter culture *P. pentosaceus*, which increases Glu (67.8 mg/100 g) and Asp (25.1 mg/100 g) levels (An et al. 2022). Additionally, *Carassius auratus* fermented with a mixed starter of *E. rivorum* and *E. lactis* shows enhanced protease activity, texture (hardness and chewiness), and flavor compounds like octanal, nonanal, and glutamic acid (Glu; Yuan et al. 2023).

## 5 Major Role of Nonlactic Microorganisms in Determining the Quality of Fermented Fish

The coinoculation of L. plantarum B8 and Staphylococcus edaphicus F from fermented mandarin fish (Siniperca chuatsi) resulted in the increased production of volatile small‐molecule compounds, including D‐limonene, phenylethanol, and anisole, which have demonstrated antioxidant, antibacterial, or anti‐inflammatory properties. The inoculated microbial communities reduced the TVB‐N, TBARS, and BAs by 25.53%, 62.82%, and 27.91%, respectively, in traditional fermented mandarin fish. The presence of this volatile compound during fermentation enhanced textural properties and safety and also improved nutritional value (Yang et al. 2025). According to Majumdar and Gupta. (2020), it was reported that seven *Staphylococci* spp. were isolated from fermented fish *sheedal* product and analyzed using NCBI‐BLAST based on rpoB gene sequences. The isolates were identified as *S*. *piscifermentans*, *S*. *condimenti*, *S*. *arlettae*, *S*. *sciuri*, *S*. *warneri*, *S*. *nepalensis*, and *S. hominis* and they exhibited moderate to high proteolytic and lipolytic activities. They were resistant to cotrimoxazole but sensitive to ampicillin, streptomycin, vancomycin erythromycin, and norfloxacin. *Staphylococcus condimenti* (ATCC 27848) was isolated from the Thai fermented fish product (kung‐chom) and identified based on phenotypic characteristics and 16S rRNA gene sequence analysis. The isolated exhibited the highest lipolytic activity when cultivated in Tween 20, 80, and lard, with activities of 6.287 ± 0.159, 5.939 ± 0.119, and 5.996 ± 0.136 U m/L, respectively, and contributed to flavor development and quality enhancement (Yiamsombut et al. 2022). Kanjasn and Sakpetch (2020) reported that *Staphylococcus simulans* PMRS35 isolated from fermented fish Budu was reported to have significant antibacterial activity against *S. aureus* under simulated gastrointestinal tract conditions, with a survival rate of more than 80%. The isolated strain exhibited sensitive to antibiotics, namely, vancomycin, erythromycin chloramphenicol, and gentamycin. *S. simulans* PMRS35 also exhibited high levels of auto‐aggregation (34.2 ± 1.4) and cell surface hydrophobicity (39.14%). The strain *Staphylococcus xylosus* isolated from Chinese fermented fish (Suanyu) was combined with mixed starter cultures (*L*. *plantarum* and *S*. *cerevisiae*) for fermentation of fish surimi. It was further demonstrated that it increased the lag time of fermentation, improved the overall acceptability and enhanced the FAA contents of 1757 and 1765 mg/100 after 72 h of fermentation (Yufei Zhang et al. 2025).

The isolated strain TL/NA/2 *Bacillus* spp. from fermented fish teles (Assam, India) demonstrated high probiotic potential under simulated gastrointestinal conditions (pH 2.0, 2.0% bile salts), achieved survival rates of 50%, autoaggregation values (↑25%), epithelial adhesion (↑80%) and improved the quality and health benefits of fermented fish. The GC–MS profiles analysis further confirmed the presence of main metabolites responsible for development of aroma and flavor, namely, oxalic acid, pyrazine, and heptane (Borthakur et al. 2025). *Bacillus* spp. is widely considered the main microorganisms responsible for alkaline fermentation in traditional fermented fish products. The *Bacillus* spp. strains, that is, 3M3A, 3M2G, 6M1C, 6M2A, 12M1F, and 12N3A isolated from Malaysian fermented fish sauce (Badu) exhibited esterase activities ranged from 118.69 ± 18.65 to 155.48 ± 15.27 U, while glutamic acid concentrations ranged from 902.33 to 1993.02 µmol/L. Furthermore, these *Bacillus* strains demonstrated probiotic activity due to their ability to produce a variety of enzymes such as lipopeptides, proteases, and lipases, which enhanced the taste, nutritional value, and safety of fermented food (Lestari et al. 2024). *B. subtilis* B‐2 was used as a starter culture for Yulu fish fermentation. The fermented product exhibited enhanced volatile compounds such as aldehyde (3‐methylbutyraldehyde, hexanal, and 2‐methylbutyraldehyde), alcohol (octanol, 1‐heptanol, and 1‐nonanol) and higher levels of 1‐penten‐3‐ol and 1‐octen‐3‐ol. In addition, the fermentation of yulu fish with *B. subtilis* B‐2 significantly reduced microbial contamination against *Pseudomonas* (31.34%–2.62%), *Achromobacter* (14.80%–0.91%), *Stenotrophomonas* (12.25%–0.85%), *Cyanobium* (12.46%–0.19%), *Rhdococcus* (9.85%–0.54%), and *Brucella* (4.51%–0.18%; Li et al. 2023).

Pla‐ra, a traditional Thai fermented fish, depended on protein and carbohydrate sources and proteolytic bacteria for enhanced taste development. Proteolytic bacteria isolated from fermented fish, such as Gallicola spp., Proteiniclasticum spp., and Bacillus spp. contributed to the production of glutamyl and arginyl peptides, indicating that these bacteria could be used as starter cultures to increase fermentation in fermented fish products (Phuwapraisirisan et al. 2024). Similarly, Det‐Udom et al. (2023) reported that fermented fish pla‐ra, analyzed using GC–MS‐based metabolomics combined with multivariate analysis, exhibited volatile metabolite profiles dominated by organic acids, particularly butanoic acid, butanoate esters, aldehydes and several sulfur‐containing compounds, notably dimethyl disulfide and dimethyl trisulfide, which were the most abundant metabolites in pla‐ra samples.

## 6 Bioactive Peptides in Fermented Fish

BPs are short chains of amino acids, typically comprising 2–20 residues and having molecular weights under 6000 Da, characterized by distinct N‐terminal and C‐terminal structures (Zhang et al. 2023b). Fermentation enhances nutritional value, decreases antinutrients, and diversifies food products by transforming raw components into beneficial compounds such as enzymes and organic acids (Tan et al. 2020; Venegas‐Ortega et al. 2019). Currently, the majority of BPs are sourced from fermented food products and synthesized by LAB, *Bacillus* spp., yeasts, and *Aspergillus* spp. There is also growing interest in exploring other potential sources of BPs (Akbarian et al. 2022). Fish fermentation generates γ‐aminobutyric acid (GABA), with L10–11 yielding the highest levels. Higher concentrations of monosodium glutamate (MSG) enhance GABA content, boosting the nutritional value of functional fermented foods (Tanamool et al. 2019). Fish protein hydrolysates (FPHs), produced by hydrolyzing fish muscle protein, contain a high concentration of BPs that exhibited multiple beneficial activities like antioxidant, antimicrobial, ACE inhibition, calcium‐binding, DPP‐IV inhibition, immunomodulatory, and antiproliferative effects (Chai et al. 2020). In *Miichthys miiuy* hydrolysate, the antioxidant peptides such as GFYAA, FSGLR, FPYLRH, VPDDD, FYKWP, FTGMD, GFEPY, YLPYA, FPPYERRQ, and GIEWA (Table 2) were identified through chromatography and RP‐HPLC. The presence of these aromatic peptides such as Ala (A), Trp (W), Phe (F), Tyr (Y), and Leu (L) enhance radical scavenging via proton donation and stabilization. while hydrophobic residues promote interaction with lipid radicals which are known for antioxidant activity (Zhao et al. 2018). The BPs isolated from fermented fish *Badu* were characterized by a high content of hydrophobic amino acids such as Leu, Ile, and functional residues such as His, and Asp which enhanced radical scavenging, metal chelation, and peptide stability, thereby contributing to high antioxidant activity. HPLC–MS/MS analysis of peptides from fermented fish *Badu* revealed novel antioxidants AIPPHPYP and IAEVFLITDPK; the latter showed significant antioxidant activity with IC_50_ values of DPPH (0.897 mg/mL) and ABTS (0.594 mg/mL; Najafian and Babji 2019). The BPs from fermented surimi (FS), analyzed by Q‐TOF‐ESI mass spectrometry, were identified as Val‐Ala‐Ser‐Val‐Ile (VSAVI, 488.32 Da), Trp‐Tyr‐Lys (WYK, 496.25 Da), and Ile‐Val‐Asp‐Arg (IVDR, 502.30 Da). The BPs from FS containing amino acids such as His, Arg, and Asp enhances activity through metal chelation and electron and demonstrated exert antihypertensive effects primarily by inhibiting angiotensin‐converting enzyme (ACE; PDB ID: 1O86; J.‐Y. Oh et al. 2019). The BPs from fermented shrimp paste, like Trp‐Pro and Ile‐Phe, displayed antioxidant activity with IC_50_ of 17.52 ± 0.46 µM, while Ser‐Val and Ile‐Phe demonstrated ACE inhibitory activity with IC_50_ values of 60.68 ± 1.06 µM and 70.03 ± 1.45 µM (Kleekayai et al. 2015). In *Katsuwonus pelamis*, the BPs such as AEM (Ala–Glu–Met), QDHKA (Gln–Asp–His–Lys–Ala), YEA (Tyr–Glu–Ala), AEHNH (Ala–Glu–His–Asn–His), and YVM (Tyr–Val–Met) showed high DPPH radical scavenging with EC_50_ values between 0.233 and 0.334 mg/mL. The BPs composed of aromatic amino acids and hydrophobic such as Val, Met, His, Ala, and Tyr enhanced the bioactivity of antioxidant peptides via enhanced peptide‐radical interactions and lipid solubility, thereby strengthening their antioxidant activity (Wang et al. 2022b). Similarly, skipjack tuna heads yielded BPs WMFDW, WMGPY, and EMGPA, which had strong scavenging activity with EC_50_ values of 0.31, 0.33, and 0.46 mg/mL (Zhang et al. 2019). Lastly, optimized hydrolysis of the scallop (*Argopecten irradians*) mantle via neutral protease and *Bacillus licheniformis* CICC 20033 produced the peptide Ser‐Val‐Pro‐Lys‐Thr‐Ala‐Thr‐Leu‐Asp‐Lys‐Tyr‐Arg (1378.1 Da), which exhibited potent ABTS scavenging activity with an EC_50_ of 2.75 ± 0.09 µM TE/µmol. The antioxidant activities of these peptides demonstrated that protein hydrolysates isolated exhibited high radical scavenging ability (Zhi et al. 2022).

**TABLE 2:** Synthesized bioactive peptides from fermented fish.

Fish (substrate)	Microorganisms (starter culture)	Types of fermentation	Fermented fish products	Synthesized peptides	References
Freshwater fish	*Lactobacillus plantarum* IFRPD P15 strain	Traditional	Pekasam	IAEVFLITDPK, AIPPHPYP	Najafian and Babji (2018)
Skipjack tuna head	Spontaneous fermentation	Traditional	Fish hydrolysate	VEE, WMFDW, DAGPYGPI, WMGPY, ERGPLGPH, EMGPA	Zhang et al. (2019)
*Cyprinus carpio*	Spontaneous fermentation	Traditional	Suanyu	GYSSYK, LYSDSK, TRTKASY	Ma et al. (2026)
*Engraulis japonicus*	*Tetragenococcus halophilus*	Traditional	Fermented fish sauce	U6‐RDEDLAP, U10‐EPAEREFEFI, U18‐PDEWEVAR, B11‐LAGICFV, B26‐IGVNLTFF, B68‐KTGPDPIPP	Han et al. (2023)
Fresh anchovies	Spontaneous fermentation	Traditional	Badu	LDDPVFIH, VAAGRTDAGVH	Najafian and Babji (2019)
Miiuy croaker	Spontaneous fermentation	Traditional	*Miichthys miiuy*	FSGLR, FPYLRH, VPDDD, FYKWP, FTGMD, GFEPY, YLPYA	Zhao et al. (2018)
Frozen Alaska pollock	Spontaneous fermentation	Traditional	Surimi	IVDR, WYK, VSAVI	Oh et al. (2019)
Skipjack tuna roes	Spontaneous fermentation	Traditional	Fish hydrolysate	AEHNH, QEP, QAEP, YVM, SGE, VDTR, AEM, TVM	Wang et al. (2022b)
*Sardinelle* muscle	*B*. *subtilis* A26, *B*. *amyloliquefaciens* An6	Traditional	Fish hydrolysate	NVPVYEGY, ITALAPSTM SLEAQAEKY, GTEDELDKY	Jemil et al. (2016)
Anchovy	Spontaneous fermentation	Traditional	Anchovy sauce	DGGP, GCK, NHP, PK	Kim et al. (2016)
Minnows/carps	*Lactobacillus acidophilus*	Traditional	Bekasam	Tyr‐Val‐Ala‐Glu; Gly‐Phe‐Pro‐Thr‐Gly‐Gly	Rinto et al. (2017)

## 7 Indigenous Fermented Fish‐Based Products in Different Parts of the World

Indigenous fermented fish‐based products across various regions illustrate a diversity shaped by unique raw materials, climates, microbial species, and dietary customs. Most traditional methods originate from Southeast Asia, Northern Europe, and Western Africa. Due to this variety, scientific research on fermented fish remains limited. Table 3 summarizes of various traditional fermented fish and fish‐based products from around the world, detailing fish species, substrate, ingredients, production methods, and treatment times, without further elaboration in the text.

**TABLE 3:** Indigenous fermented fish products worldwide.

Common name	Substrate	Production steps	Treatment time	Products	Bacteria isolated	Country	References
Tungtap	*Puntius* spp. or *Danio* spp.	Washing; drying: 3–4 days; room temperature; Pre/postsalting (1:10% w/w)	3–6 months	Whole fish	*Lactobacillus* spp., *Enterococcus* spp., *Streptococcus faecalis, Enterococcus* spp., *Staphylococcus aureus*, *S. saprophyticus*	India	Joshi et al. (2020)
Shidal	*Puntius saphore*; *Setipinna phasa*	Washing; drying; postsalting (1:10% w/w); ambient temperature	3–5 months	Whole fish	*L. plantarum*, *P*. *lolii*, *P. pentosaceus*, *P*. *acidilactici*	India	Gupta et al. (2021)
Utonga‐kupsu	*Esomus danricus*	Pre/postsalting; room temperature	20–25 days	Whole fish	*Staphylococcus piscifermentans*, *S*. *condimenti, S*. *carnosus*	India	Singh et al. (2018)
Plaa‐som	*Barbodes gonionotus*	Presalting (8:1, w/w); substrate mixture (minced garlic, boiled rice); room temperature	1 week	Whole fish	*Pediococcus pentosaceus*; *Zygosaccharomyces rouxii*	Thailand	Nicomrat et al. (2018)
Nam‐pla	*Osteochilus hasseltii*	Pre/postsalting; ambient temperature	12–18 months	Sauce	*Fusobacterium* spp.; *Peptostreptococcus* spp.; *Gallicola* spp.; *Halanaerobium* spp.	Thailand	Khongla et al. (2020)
Badu	*Sardinella* spp.	Presalting; 30–40°C	6–12 months	Fish sauce/paste	—	Malaysia	Hajaratul Najwa Mohamed et al. (2012)
Bakasang	Cakalang fish	Presalting; 30–50°C	7 days	Fish sauce/paste	*Pediococcus* spp.; B5.1, *P*. spp. B1.0	Indonesia	Lawalata and Rungkat (2019)
Yu‐lu	Anchovies	Prefermentation salting (1:3%, w/w); 20–25°C	2–6 months	Fish sauce	*Firmicutes*; *proteobacteria*; *Halanaerobium*	China	Wang et al. (2018)
Suanyu	Carp	Washing; gutting; salting (6%–12% w/w); anaerobic fermentation	1–2 months	Whole freshwater fish	*Lactobacillus; Tetragenococcus; Weissella*	China	Liuet al. (2021)
Zhayu	Grass carp	Presalting	15–30 days	Whole fish	*L. plantarum; P. acidilactici; P. Pentosaceus*	China	An et al. (2022)
Anchovy fish sauce	Fresh anchovies	Washing; mixing anchovies with solar salt (∼30% w/v); ambient temperature	15 months	Fish sauce	*Tetragenococcus; Carnobacterium; Gallicola*	China	Du et al. (2019)
Chaoshan fish sauce	Anchovy	Presalting	6–18 months	Whole fish	Carnobacterium; Lentibacillus	China	Ma et al. (2022)
Paste (Yucha)	Silver carp	Presalting	10–20 days	Fish flesh	*L. plantarum; L. lactis*	China	Han et al. (2020)
Mandarin fish	*Siniperca chuatsi*	Washing; immersion in 6% brine (1:1.5, w/v); natural or starter inoculation; controlled fermentation	15–20 days	Whole fish	*L. Sake; L. plantarum; P. platycococcus*	China	Guo et al. (2025)
Jeotgal	Anchovy (*Engraulis japonica*)	Presalting (5%–30%, w/w); 10–30°C	2 months	Whole fish	*Lactobacillus sakei*; *L*. *curvatus*; *Weissella koreensis*	Korean	Song et al. (2018)
Katsuobushi	*Katsuwonus pelamis, Euphonia affinis*	Presalting (10%–15% w/w); ambient temperature	3–4 months	Whole fish	*Aspergillus*; *A. chevalieri*; *A*. *pseudoglaucus*; *A. sydowii*	Japan	Takenaka et al. (2020)
Narezushi	Crucian carp	Presalting (20%–30%, w/w)	2–3 months	Whole fish	*Lactobacillus acidipiscis*; *L. versmoldensis*	Japan	Kuda (2017)
Rakfish	Trout or char	Gutting; salting 4%–7% (w/w); brine soaking	3–12 months	Whole fish	*Psychrobacter*; *Lactobacillus*	Norway	Bjerke et al. (2019)
Hákarl	*Somniosus microcephalus*	Chopping; cleaning; dry	3–4 weeks	Whole fish	*Tissierella creatinophila*	Iceland	Osimani et al. (2019)
Surströmming	Atlantic herring	Immersion; Decapitation; dissection; 15—18°C	4–12 weeks	Whole fish	*Carnobacterium* spp.; *H. praevalens*; *T. halophilus*; *Clostridiisalibacter* spp.	Sweden	Belleggia et al. (2020)
Enam Ne–Setaakye	*Alestes nurse, Tilapia nilotica*	Presalting; ambient temperature	72 h	Whole fish	*Lactobacillus brevis*; *Pediococcus pentosaceus*	Ghana	Michael et al. (2019)
Momoni	Snakehead fish	Presalting; 14%–40% (w/w)	2–3 days	Whole fish	*Micrococcus*; *S*. *aureus*; *Lactobacillus* spp.; *Pseudomonas*; *Pediococcus*	Ghana	Bamidele et al. (2023)
Lanhouin	*Pseudotolithus senegalensis*	Presalting	3–9 days	Whole fish	*B. subtilis*; *S*. *lentus*	Ghana, Benin	Anihouvi et al. (2012), Zannou et al. (2022)
Gyagawere	Catfish, croaker, meagre, shark	Presalting	3 days	Whole fish	*Leuconostoc lactis*; *Lactobacillus fermentum* spp.	Ivory coast	Banwo et al. (2020)
Guedj, tambadiang, yet	Mackerel, seabream, threadfin, croaker, mullet, catfish	Pre/postsalting	The whole night‐ 2 days	Whole fish	*Proteus* spp.; *Shewanella*; *Bacillus* spp.	Gambia, Senegal	Akande (2013)
Fesikh	*Alestes baremose*, *Hydrocynus* spp.	Pre/postsalting; 1:20%–1:30% (w/w); ambient temperature	15 days–3 months	Whole fish	—	Egypt	Amin et al. (2020)
	*Sardinella* spp., *Micromesistius poutasssou*	Presalting (15%; w/w); ambient temperature	2 weeks	Whole fish	*Shewanella putrefaciens*; *Streptococcus faecium*; *Penicillium notatum*; *Cryptococccus laurentii*; *Torulaspora debrueckii*	Nigeria	Achinewhu and Oboh (2002), Fawole and Oyelese (2019)

### 7.1 Fermented Fish in Asia

In Asia, particularly Southeast Asia, fermented fish products are commonly found in both rural and urban areas, marketed under traditional names such as Malaysia's budu, Thailand's nam‐pla, Japan's shottsuru, Nepal's sidra, Bhutan's maacha, Myanmar's nga‐pi, and Korea's jeot (Anal et al. 2020; Molinos et al. 2016). In India, fermented fish is a significant part of the cuisine, particularly in the northeastern region. Northeast India, home to more than 100 tribal tribes, each of which has its own distinct food traditions has a long history of fermenting fish for preservation. Fermented fish products, passed down through generations, include lonailish, tungtap, shidal, hentak, and utonga‐kupsu (Pohsnem et al. 2023). Fermented fish is rich in nutrients and probiotic bacteria, especially LAB, offering various health benefits. It promotes gastrointestinal microbiota balance, metabolic syndrome, cardiovascular, and neurological health (Abrar and Jaffri 2023). Tungtap is a popular ethnic fermented fish product that is primarily prepared and consumed by the tribals of Meghalaya. To prepare Tungtap, raw fishes such as *Puntius* or *Danio* spp. are washed and left overnight to reduce moisture. They are then mixed with fish fats and salted at a 1:10 ratio. Next, the fish are placed in oiled earthen pots, sealed with a mixture of fish scales and clay oil slurry, and left to ferment at room temperature for 3–6 months (Majumdar 2022; Pohsnem et al. 2023). The LAB isolated from tungtap include cremoris, *Lc*. *plantarum*, *E*. *faecium*, *Lb*. *fructosus*, *Lb. amylophilus*, *Lb*. *coryniformis* subsp. torquens, *Lactococcus lactis* subsp., *Lb. plantarum*, *Lb*. *puhozihii*, *B. pumilus*, *Micrococcus*, and yeast species (Jhamb and Swaminathan 2023). Korea's jeotgal, or jeot, is a widely consumed fermented fish dish, with around 30 commercial varieties from over 160 types, often served with kimchi or as a side. Varieties like Sikhae, Hongeo, and Myeolchi‐Jeot are common (Lee et al. 2021; Singh et al. 2017). Sushi is a popular Japanese meal made from salted fermented fish. Historically made with freshwater fish, it now also uses marine fish due to higher demand. Fermented fish such as narezushi, is valued for its health benefits as an intestinal regulator and is traditionally eaten during autumn village festivals (Sionek et al. 2023; Yamada 2019). Indonesia, known for its high marine fish production, has developed several popular traditional fermented fish preparations, including Terasi, Peda, and Wadi (Prihanto et al. 2024). The scientific literature on Chinese fermented fish products identifies several main types. Suan Yu is a low‐salted snack that retains the nutritional value of raw fish while having a fishy taste. Yucha is a popular supplementary meal among the Li population in Hainan province. Chouguiyu is noted for its firm yet soft texture and strong odor. Zaoyu is appreciated for its appealing aroma derived from fermented glutinous rice vinasse (Belleggia and Osimani 2023). Thai fermented fish, called Plaa‐som (or Pa‐som in Laos), is made using a variety of techniques depending on the family and flavor preferences (Ly et al. 2018). Ngapi, a traditional fermented fish made from salted fish or shrimp, is an essential part of Burmese cuisine. Vietnamese fish sauce, called nuoc mam, is made by layering salt and allowing fish and shrimp to ferment (Narzary et al. 2021). In Indonesia, popular fermented fish items such as terasi, peda, and kecap ikan account for less than 2% of all processed fermented fish products (Hafifah 2023). Similarly, yu‐lu, a fermented fish sauce, is an essential condiment in Chinese cuisine. In Japan, fermented fish is commonly known as shiokara (whole or partial fish) and shiokara paste (pounded or grated). Korea has a long history of fermented foods, with popular varieties including jeotkal, eo‐ganjang, and sikhae. The “Colombo cured” process is widely used in Southern India and Sri Lanka to prepare fermented fish (Belleggia and Osimani 2023; Pohsnem et al. 2023).

### 7.2 Fermented Fish in Africa

In Sub‐Saharan Africa, fermentation has long served as an important preservation technique, enhancing food safety, extending shelf life, and improving nutritional quality. Various fermented fish products, such as adjuevan, gyagawere, jalan, bunyi, aku, youri, fessiekh, djegue, momone, guedj, terkeen, mindeshi tambadiang, yet, and salanga, are dietary staples with extended shelf lives and cultural importance (Apaliya et al. 2022). When properly handled, these products contribute to economic growth and improve the quality of life. However, conventional processing techniques often lack formalization and standardized procedures, thereby increasing the likelihood of contamination and compromising both safety and market viability (Ayeloja and Jimoh 2022). Products like adjuevan and salanga, which are widely consumed across Sub‐Saharan Africa, exemplify the dual nature of fermented fish, offering both nutritional benefits and potential risks when inadequately processed (Anumudu et al. 2024; Zang et al. 2020).

Momoni, a popular fermented fish in Ghana, is sun‐dried on wooden trays, resulting in a high salt content. Typically, it is used in stews with red pepper, tomato, onion, and palm oil. The fish, derived from African jack mackerel, is scaled, gutted, and rinsed in tap water before being heavily salted (294–310 g/kg), with particular attention paid to the gills and gut. It ferments in baskets covered with aluminum trays or jute bags for 1–5 days (Bamidele et al. 2023). Adjuevan, a salted and fermented fish from the west coast of the Ivory Coast, is prepared according to traditional methods. The fish undergoes a 5‐day fermentation process in sealed jars lined with plastic and filled with stones. After fermentation, they are dried at 28–30°C for a minimum of 10 days on racks or nets (Abré et al. 2023). Adjuevan fermented fish has been shown to contain flavor components (73.33%), pH (7), moisture content (5.31%), and protein content (68.91%), respectively. It is prone to contamination by food spoilage bacteria, including *Enterobacter* spp*., Pseudomonas* spp*., Klebsiella* spp*., Pseudomonas fluorescens*, and *E. coli*, which are the main spoilage organisms. Less frequently encountered are *Salmonella* sp.*, Serratia marcescens, Salmonella arizonae*, and *Proteus* spp. as described previously (Koffi and Koussémon 2012). On the other hand, higher levels of *Staphylococcus, Lactobacillus, Psychrobacter, Peptostreptococcus*, and *Fusobacterium* were observed to be associated with increased concentrations of biogenic compounds such as histamine, cadaverine, putrescine, and tyramine (Abré et al. 2023). Adjuevan, a specialty from the Ivory Coast, undergoes fermentation with yeast varieties such as *Kluyveromyces marxianus*, *Hansenula anomala*, *Saccharomyces cerevisiae*, *Candida tropicalis*, and *Candida*. Traditional fermentation methods regulate sodium chloride levels and yeast selection in adjuevan. Notably, yeasts like *Kluyveromyces marxianus* and *Torulaspora delbrueckii* exhibit enhanced resistance to sodium chloride, underscoring their pivotal role in adjuevan fermentation (Clémentine et al. 2020). Fesikh, a traditional dish from ancient Egypt, ferments Bouri fish (*Mugil cephalus*) using natural or modern industrial methods, resulting in its unique flavor and aroma. There are two types of feseekh available: a less‐salty variety that is ready in 15–20 days, and a more heavily salted variety that matures over 2–3 months before consumption. Industrial fermentation, with controlled salting, enhances quality by swiftly reducing microbial growth, particularly coliforms while improving color, texture, flavor, and aroma compared to natural methods (Asar 2024).

### 7.3 Fermented Fish in Europe

In Europe, fermented of foods, particularly fish, has a long tradition, although only a few varieties remain commonly produced today, such as garum, a renowned fish sauce from ancient Greece and Rome (Belleggia and Osimani 2023). Historically, fermentation has been the primary method of fish preservation worldwide. However, only a few fermented fish products are still produced in Northern Europe. This process depends on microorganisms and natural enzymes in the fish, which help develop distinct textures, tastes, and aromas, with enzymes primarily contributing to flavor (Kieliszek et al. 2021). Historical accounts suggest that during the Middle Ages, salt was less accessible, and meals often had strong, pungent aromas, making fermented fish common in Nordic countries, especially as a winter staple (Svanberg 2015). Innovations in food preservation throughout the 19th century introduced refrigeration and transportation advancements, shifting food preservation practices and reducing the reliance on traditional methods such as smoking, fermenting, and drying (Svanberg and Locker 2020; Tamang et al. 2020). Rakfisk, a Norwegian fermented fish dish, is made by salting and fermenting fishlike trout or char at low temperatures (4–8°C) for several months, resulting in a strong flavor. Traditionally, it was a popular dish from late fall through Christmas and is sometimes paired with flatbread, sour cream, and onions (Østerlie and Wicklund 2018; Skåra et al. 2015; Zang et al. 2020).

Since the Viking Era, Northern European cultures have successfully preserved food through the salting (Belleggia et al. 2020). Notably, the Scandinavian Peninsula and Iceland developed unique preservation techniques based on empirical methods due to the limited availability of salt in those regions (Skåra et al. 2015). Consequently, fish‐based delicacies with unique sensory qualities have been generated by using small quantities of salt in combination with newly developed techniques. Over the Christmas season and in late October, Norwegian rakfisk, a fermented fish, is a popular dish. Rakfisk is still produced in most of the interior parts of the nation. Rakfisk's unique flavor, aroma, and spreadable texture (Bjerke et al. 2019; Skåra et al. 2015). Surströmming is perhaps the most well‐known fermented fish dish in Europe. It is made from Baltic herring that has been brine‐fermented for a few months. Surströmming, known for its powerful scent, is typically consumed with potatoes, onions, and sometimes flatbread. The term “Surströmming” originated from the herring preservation methods used by the inhabitants of the Swedish Gulf of Bothnia coast in the sixteenth century. The earlier preparation has a strong odor and turns wine‐colored, which could make it difficult to eat (Skåra et al. 2015; Belleggia et al. 2020). In the northern regions of Sweden, surströmming, a fermented fish delicacy made from herring, is well‐known for its distinct aroma. The herring is fermented in barrels at 15–18°C for 3–4 weeks after being presalted in a saturated salt solution and having its head and stomach removed. When the fermented product has finished fermenting, it is transferred to cans together with its brine. The fermenting process in the can continues for about 6 months. The anaerobic halophile bacteria are responsible for developing the surströmming features (Belleggia et al. 2021). According to reports, the usual composition of surströmming is 11.8% protein, 8.8% salt, and 3.8% fat, with a pH of 7.1–7.4. Maatjes, a popular fish dish in the Netherlands prepared with lightly salted Baltic herring, is another application for this species. The best time to catch herring is right before it spawns, which is in May and July. Its subcutaneous fat content is ideal, ranging from 16% to 20% (Skåra et al. 2015). For generations, Icelandic people have been fermenting or curing sharks, or *Somniosus microcephalus*, known as hákarl. While neighboring nations were aware of the prevalent usage of salt for preservation, Iceland faced challenges in producing salt due to a lack of fuel. It was necessary to find other ways, and in Iceland, traditional food preservation techniques included drying, fermenting (kaesing), and acidifying food with lactic acid (milk whey; Skåra et al. 2015). Shark fishing gained popularity in Iceland in the fourteenth century. Hakarl was a staple of the Icelandic diet for many centuries. Hakarl tastes solidly like fish, looks cheesy, smells strongly of ammonia, and has a soft texture (Jensen et al. 2023; Skåra et al. 2015). Because of its richness and ubiquity, Baltic herring is one of the most emblematic fish of the Baltic Sea. Herring and mackerel are considered the best ingredients for garum. The fermentation process lasted at least 9 months. Several small Italian and Spanish companies still utilize the same recipe for garum to manufacture their fish sauces (Mouritsen and Styrbæk 2020). Popular in Iceland, hakarl is a fermented shark flesh delicacy that has been kept well for years (Isola et al. 2022). Only the two procedures fermentation and drying can produce it. Shark fermentation can take 3–6 weeks, while drying times can range from a few weeks to a few months, depending on the weather and fishing season. During fermentation, the bacteria also converted trimethylamine N‐oxide (TMAO) to TMA, and the ureases in hakarl converted urea to ammonia. Nonetheless, there was a slight drop in TMA and ammonia levels throughout the drying process (Skåra et al. 2015). Additionally, this potentially hazardous raw material, fresh shark meat, which is thought to be deadly, may be turned into a wholesome meal. Fermented hakarl was an important source of energy and nutrition for Icelanders in the past and is still a favorite drink among the elderly today. However, relatively few conventionally fermented fish products are produced. For instance, the well‐known fermented fish sauce garum is attributed to the Greeks and Romans. The Norwegian people have been consuming herring (*Clupea harengus*) for over a millennium. Remains of herringbones found during excavations show how important this fish is as a dietary source (1159–1181; Eliasen et al. 2021; Kyselý et al. 2022).

## 8 Various Health Benefits and Safety of Fermented Fish

Fermentation, primarily used for preservation, also enhances flavor, digestibility, and therapeutic properties in fish. Research indicates that fermented fish holds significant potential in functional foods and pharmaceuticals due to its rich content of vitamins, FAAs, and minerals, making it an effective nutritional supplement in areas affected by malnutrition (Marti‐Quijaet al. 2020). Probiotics in fermented fish benefit various body areas, including the gastrointestinal tract, mouth, urinary and respiratory tracts, and the vagina, with a primary focus on gastrointestinal health across diverse populations. These probiotics support digestion by breaking down complex carbohydrates, maintaining colon pH, and inhibiting harmful bacteria (Barajas‐Álvarez et al. 2023). Fermented fish contains LABs such as *L. plantarum* and *Saccharomyces cerevisiae*, which contribute to various health benefits, including reduced inflammation, enhanced immunity, and symptom relief for pain and constipation. These probiotics have also shown positive immune effects in HIV‐positive children, promoting overall well‐being (Chilton et al. 2015; Diez and Astiazaran 2022). Consuming fermented fish has been associated with cancer prevention, antimicrobial properties, and immune modulation. LABs in these products may positively alter gut microflora, reduce cancer risk by lowering beta‐glucuronidase, and improve bile salt tolerance and cholesterol levels. Furthermore, probiotics help balance gut microbiota to regulate immune responses, preventing inflammation linked to cancer (Khaledi et al. 2023; Şanlier et al. 2019). Fermented fish products have been reported to provide several biological benefits, such as antioxidant, intestinal health, inhibitory effect, anticancer, and antihypertensive activities (Figure 6).

### 8.1 Antioxidant and Antimutagenic Properties

**FIGURE 3:**
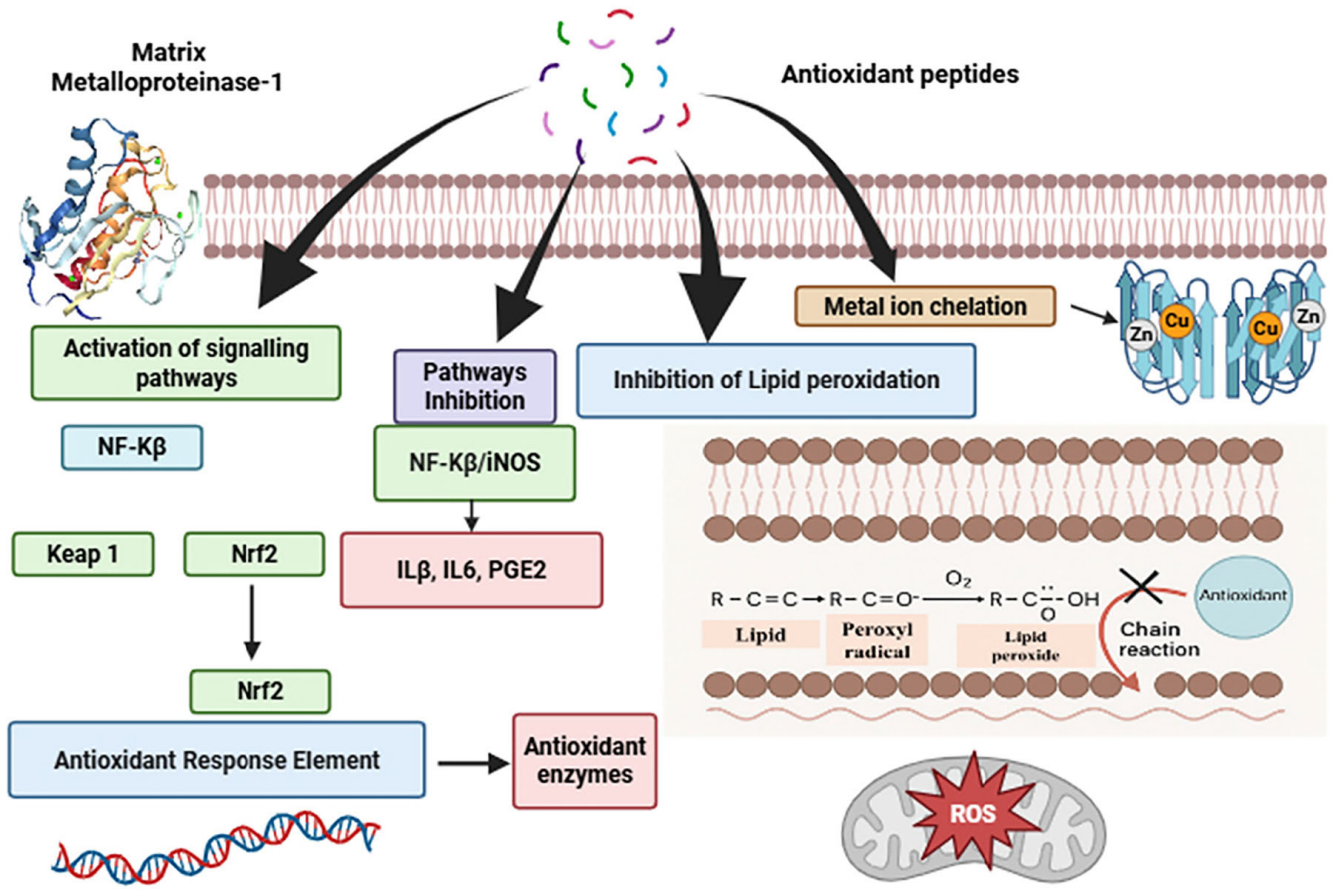
Schematic representation of antioxidant peptide‐mediated inhibition of lipid peroxidation and modulation of NF‐κB/Nrf2 signaling pathways.

Oxidative stress arises primarily from excess free radicals generated by unhealthy lifestyle factors including the consumption of processed foods, tobacco use, and exposure to industrial chemicals, pollutants, and radiation. These reactive species can damage cellular membranes, enzymes, proteins, and DNA, thereby increases the risk of hypertension, diabetes, cancer and cardiovascular diseases. In vitro studies indicate that antioxidant peptides derived from fermented fish can inhibit lipid peroxidation and modulate NF‐κB/Nrf2 signaling pathways, as illustrated in Figure 3. Fermentation can enhance antioxidant extraction and alter antioxidant profiles in foods, creating bioactive compounds with health benefits (Adebo and Gabriela 2020). LABs are primary microbes in bacterial fermentations and act as starters for producing fermented fish, Their antioxidant activity has mainly been demonstrated using in vitro chemical assays, such as DPPH and ABTS radical scavenging methods (Khubber et al. 2022). In animal by‐products, antioxidants mainly derive from BPs formed via fermentation or enzymatic protein hydrolysis. Fermentation also releases new antioxidant compounds such as exopolysaccharides, produced by microorganisms produce from sugars, while protein‐rich fermented foods yield BPs with beneficial antioxidant properties against oxidative stress. These peptides are produced by starter culture enzymes during protein hydrolysis, thereby enhancing, antioxidant activity in fermented fish. In food production, antioxidants naturally preserve processed foods by inhibiting oxidation. An in vitro investigation on Loma fermented fish extract demonstrated the presence of BPs capable of scavenging free radicals, as measured by the DPPH assay. The radical‐scavenging activity was 68.81 ± 0.76%, with an IC_50_ value of 1.36 mg/mL. The results highlighted the potential role of BPs such as Ala‐Ile‐Pro‐Pro‐His‐Pro‐Tyr‐Pro and Ile‐Ala‐Glu‐Val‐Phe‐Leu‐Ile‐Thr‐Asp‐Pro‐Lys as sources of natural antioxidant ingredients, relevant to food preservation and pharmaceutical industries (Najafian and Babji 2018). The antioxidant peptides isolated from the Budu were analysis by HPLC and ESI‐TOF MS/MS and the results reported the presence of amino acid such as Ile, Leu, Asp and His in the peptide sequences contributed to enhanced antioxidant activity of the fermented fish. Therefore, the extracts peptides could potentially use as antioxidant sources in nutraceutical product. Among the different BF extracts, BF‐III exhibited the highest ABTS radical scavenging, with an IC_50_ value of 0.544 ± 0.31 mg/mL. In contrast, BF‐II, BF‐I and Budu demonstrated higher IC_50_ values of 0.841 ± 0.08, 2.14 ± 0.43 and 1.25 ± 0.15 mg/mL, respectively (Najafian and Babji 2019). Fermented fish by product from keropok lekor was utilized as substrate and *Lactobacillus casei* strains (LC216, LC217, LC219, and LC220) were employed to convert these byproducts into bioactive FPHs. The production of bioactive FPHs in the fermented products exhibited higher radical scavenging activity (82.8%–88.4%) compared with conventionally fermented fish products (78.9%). Thus, these bioactive FPHs could potentially use as bio‐ingredient in nutraceutical and functional foods application (Abd Rashid et al. 2022). Extracts from fermented fish have demonstrated high antioxidant activity, attributed to the presence of bioactive compounds that may promote protection, prevention, or reduction of oxidative stress–related effects. In vitro study, punti extract exhibited the highest antioxidant activity (80.15 ± 5.67%) compared with shidal extract, which showed an activity of 68.30 ± 3.22% (Majumdar et al. 2016b). In vitro studies, the *Lactobacillus brevis* LAP2 strain isolated from traditionally fermented hentak fish exhibited notable antioxidant, antibacterial, and probiotic benefits, with activity from 18.8 ± 0.65% to 68.35 ± 0.64%. The isolated strain *L. brevis* LAP2 was analyzed and reported to exhibit probiotic potential and could be used in pharmaceuticals and food industries (Aarti et al. 2017). In addition, Yong and Yu published a literature review on in vitro studies of DPPH scavenging activity of frequently consumed fermented fish products. Most products, including sikhae, exhibited activity above 50%, anchovy sauce (69.2%), pekasam (68.81%), jeotgal (63.83%), and shrimp bagoong (61.5%). Among all products, round scad sauce had the highest activity rate at 83.5% (Cha and Yu 2024). In vitro studies, the DPPH radical‐scavenging activity of extracts from *Utonga‐kupsu*, *Hentak*, and *Ngari* exhibited inhibition values of 40.53%, 35.77%, and 52.11%, respectively (Singh et al. 2018).

Fermented fish products offer significant antioxidant and antimutagenic benefits. Probiotics can influence immunity via gut‐associated lymphoid tissue and produce butyric and acetic acids, which possess antimutagenic properties. Antimutagens counter DNA mutations by affecting replication, repair, or neutralizing mutagens through chemical or enzymatic actions (Pop et al. 2022). Gut bacteria like *Bifidobacteria* can bind and reduce carcinogen mutagenicity, enhancing their probiotic dietary value (Alessandri et al. 2019). For instance, *L. acidophilus* and *Bifidobacterium* species provide antimutagenic effects (Chandel et al. 2019). *L. plantarum*, isolated from fermented durian (*Durio zibethinus*), exhibited high antimutagenic effects against sodium azide (NaN3) and 2‐aminoanthracene (2‐AA) by binding to mutagens and preventing mutagenesis. In vitro studies the presence of metabolic activation, the antimutagenic activity against *Salmonella typhimurium* TA98 and TA100 were 93% and 50%, respectively, for bacterial cells, and 91.7% and 36%, respectively, for the cell‐free supernatant (CFS; Ahmad et al. 2018). According to Koo et al. (2016), fermented fish meyolchijeot shows antimutagenic activity of 26.6%–43.4% after 6–12 months of fermentation. However, evidence supporting antimutagenic effects in vivo or in human populations remains limited, highlighting the need for further investigation.

### 8.2 Anticancer Activities

**FIGURE 4:**
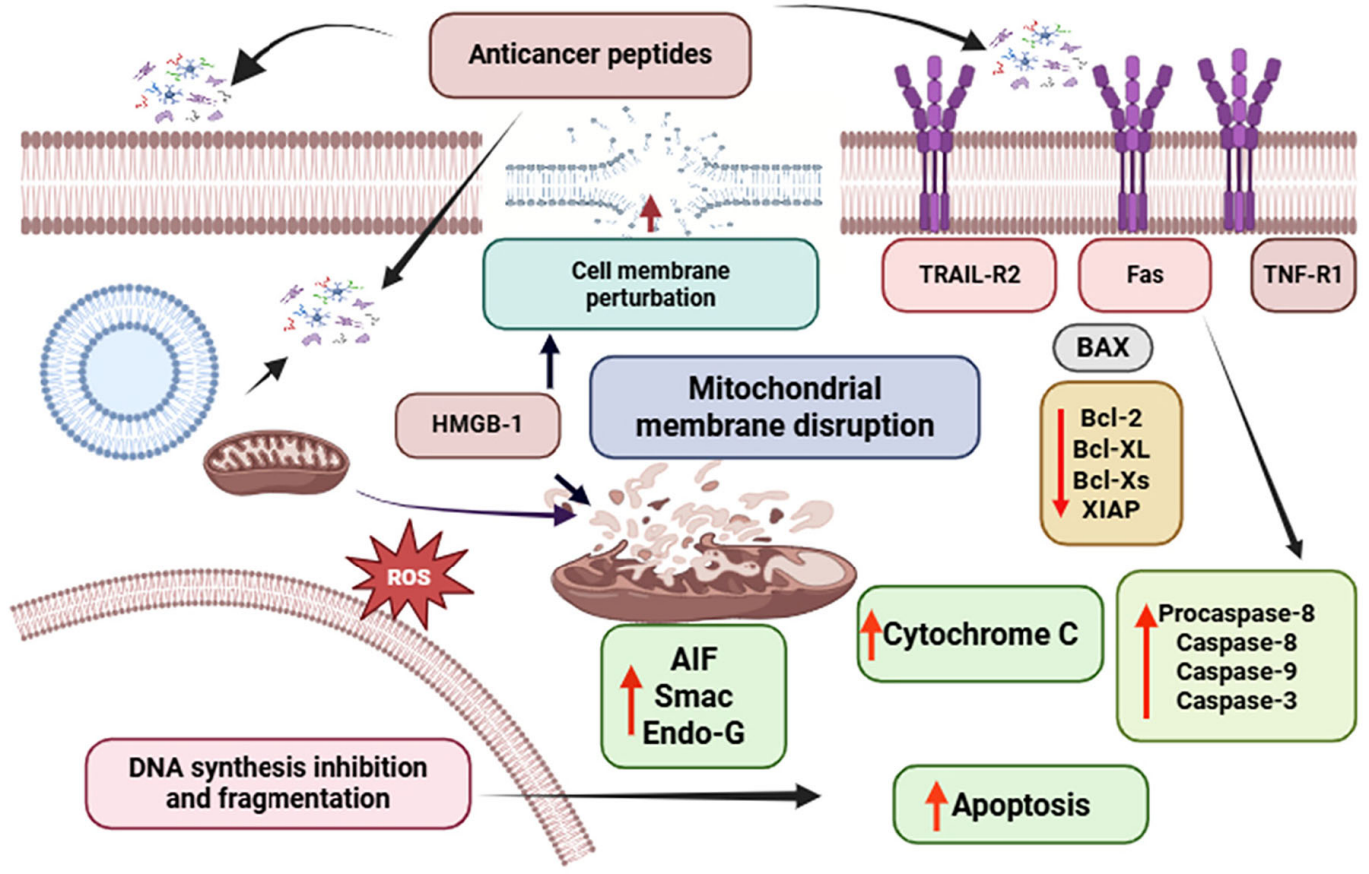
Schematic representation of anticancer peptide‐induced mitochondrial dysfunction and activation of caspase‐mediated apoptotic pathways.

Fermented fish's antioxidant activity may help reduce oxidative damage, which is a known contributor to carcinogenesis. BPs found in protein‐rich fermented fish products have demonstrated notable anticancer potential primarily in in vitro models. Peptide fractions derived from anchovy sauce extracts, for example, induced apoptosis in cancer cell lines under controlled laboratory conditions (Kumari et al. 2022). The mechanism of anticancer peptide‐induced mitochondrial dysfunction and activation of caspase‐mediated apoptotic pathways is shown in Figure 4. According to Ibrahim et al. (2023), in vivo investigation indicated that specific strains of *Lacticaseibacillus casei* YIT9029 and *Bifidobacterium longum* can metabolize and neutralize mutagenic substances and inhibit cancer cell proliferation. BPs isolated from anchovy, kajami‐sikhae, and jeotgal exhibited strong inhibitory activity against the HepG2 liver carcinoma cell line, exerting anticarcinogenic effects through apoptosis induction related to somatic mutations (Koo et al. 2016). Probiotics isolated from Utonga‐kupsu fermented fish such as *Staphylococcus* spp., *S. carnosus*, and *S. piscifermentans* exhibited significant cytotoxicity against HeLa and HT‐29 cancer cell lines, without affecting normal lung L‐132 cells in vitro studies. The presence of such microorganisms could represent a potential source of probiotic strains and may contribute to cancer preventive effect (Singh et al. 2018). However, these effects have not yet been validated in human clinical trials. The isolated of protein hydrolysate from fermented fish sauce (Nam‐pla) exhibited cytotoxic effects on HepG2 liver cancer, with cell viability ranging from 106.81 ± 7.07% to 112.51 ± 4.27% when treated with 200–400 µg/mL concentrations for 72 h. This result revealed the possible application of the BPs from fermented fish sauce as natural antibiotics (Khositanon et al. 2018). In vitro studies, an active compound from the supernatant of *Streptomyces parvus*, a marine organism, exhibited anticancer effects on HepG2, EI‐4, and MCF‐7 cells, with EC_50_ inhibition rates of 53%, 56%, and 57%, respectively (Abd‐Elnaby et al. 2016). Finally, in vitro studies, fermented fish sausages containing probiotic *Enterococcus* spp. (MF067470, MF067509, and KY96290) isolated demonstrated cytotoxic effects against the Caco2 colon cancer cell line (18.0%–24%) and the MCF‐7 breast cancer cell line (13.9%–27.9%), without affecting normal cells under experimental conditions (AlKalbani et al. 2019). Han et al. (2023) reported that hydrophobic peptide extracts from fermented fish sauces, such as kajami‐sikhae and chuneobamjeot, exhibited anticancer activity by induced of apoptosis in a human lymphoma cell line (U937) and could be regarded as potential anticarcinogenic agents due to their strong antiproliferative effects.

### 8.3 Antimicrobial Activities and Improvement of Gut Microbiota

**FIGURE 5:**
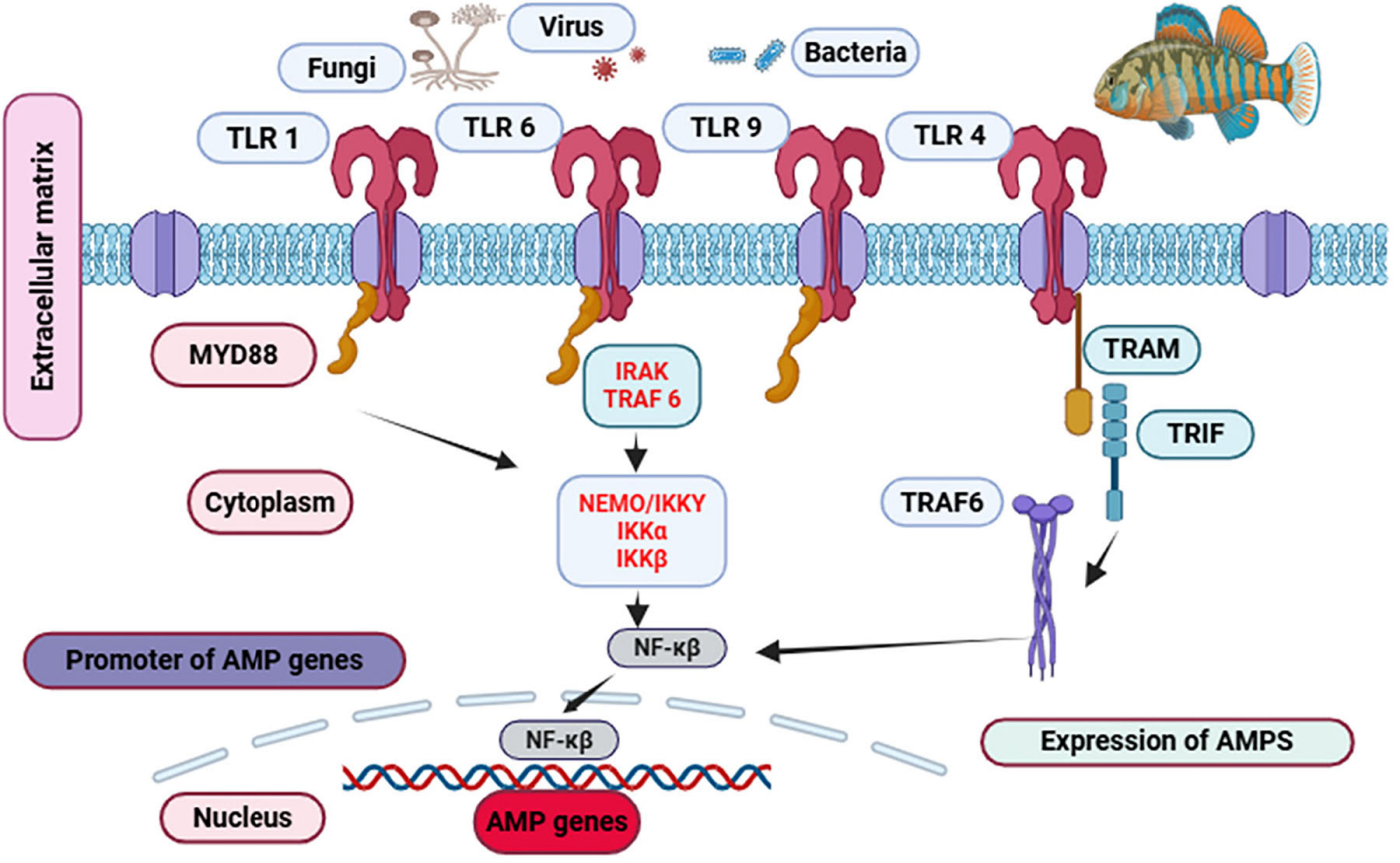
Schematic representation of toll‐like receptor (TLR)‐mediated signaling pathways regulating antimicrobial peptide (AMP) gene expression in fish. LABs from pekasam fermented fish, including *Lactobacillus plantarum*, *L. pentosus*, and *Lactococcus lactis*, show inhibitory activity against pathogenetic microorganisms such as *Klebsiella* spp*., S. aureus*, and *E. coli*, while tolerating high pH levels (3.0 and 5.0) and 0.3% bile. These probiotics support microbial diversity, protect the gut lining, and improve GI health (Ida Muryany et al. 2017).

Certain LABs isolated from fermented fish exhibit antimicrobial effects against bacteria, yeast, and filamentous fungi, making them effective natural preservatives. These antimicrobial properties primarily derive from LAB metabolites, including bacteriocins, lactic acid, H_2_O_2_, and organic acids. LAB strains like *Enterococcus*, *Leuconostoc*, *Lactobacillus*, *Pediococcus*, and *Weisella*, among others, are prevalent in traditional Indonesian fermented foods (Aleksanyan et al. 2024; Amarantini et al. 2019). LAB can damage the outer membranes of pathogenic bacteria (gram ± pathogens), resulting to cell lysis, while bacteriocins, ribosome‐produced antimicrobial peptides, form pores in bacterial membranes. Bacteriocins show bactericidal or bacteriostatic properties against food spoilage pathogens (Mahmoud and Saber 2022). The mechanism of toll‐like receptor (TLR)‐mediated signaling pathways regulating antimicrobial peptide (AMP) gene expression in fish is shown in Figure 5 . The antimicrobial properties of fermented fish products stem from LAB‐produced peptides during fermentation; yeast, *Bacillus* spp., and specific fungi are typically added as fermentation starters to release antimicrobial peptides (Chourasia et al. 2023). For instance, in vitro studies LAB isolated from fermented fish peda exhibited a strong inhibitory effect against *S. aureus* ATCC 25923 (18.7 ± 2.1 and 18.0 ± 2.7 mm) and *Salmonella typhi* BPE 122.4.CCA (19.5 ± 0.3 and 19.0 ± 0.7 mm). The antimicrobial analysis reported that the two isolated strains produced bacteriocins LAB Pr.3.4L and Pi.5.8 (Amarantini et al. 2019). Six LABs (IB1C, IB3B, IB3C, IB3E, IB3F, IB6C) were isolated from fermented fish *chao* exhibited inhibition zones against *S. aureus* FNCC0047 ranged from 18.67 ± 0.58 to 16.50 ± 2.29 mm while *E. coli* FNCC0049 ranged from 14.67 ± 1.15 and 9.33 ± 0.57 mm in vitro studies. Therefore the isolated strain identified as *L. plantarum* by gene sequences could be used as preservative inoculum for controlling contamination in food products (Nurhikmayani et al. 2019). *P. acidilactici* isolated from fermented fish bekasam demonstrated antimicrobial activity against the pathogenic bacteria with inhibition zones of 5.10, 21.26, and 18.23 mm against *L. monocytogenes* CFSAN004330, *E*. *coli* O157, *S*. *aureus* ATCC 25923, respectively (Purwati et al. 2019). Similarly, *Aerococcus* NJ‐20 was isolated from peda, exhibited inhibitory activity against *S. aureus* ATCC 6538 with an of 7.6 ± 1.35 mm inhibition zone in vitro *studies*. The results indicated that the inhibition of the pathogenic bacteria was due to the acidification of the medium (Putra et al. 2018).

**FIGURE 6:**
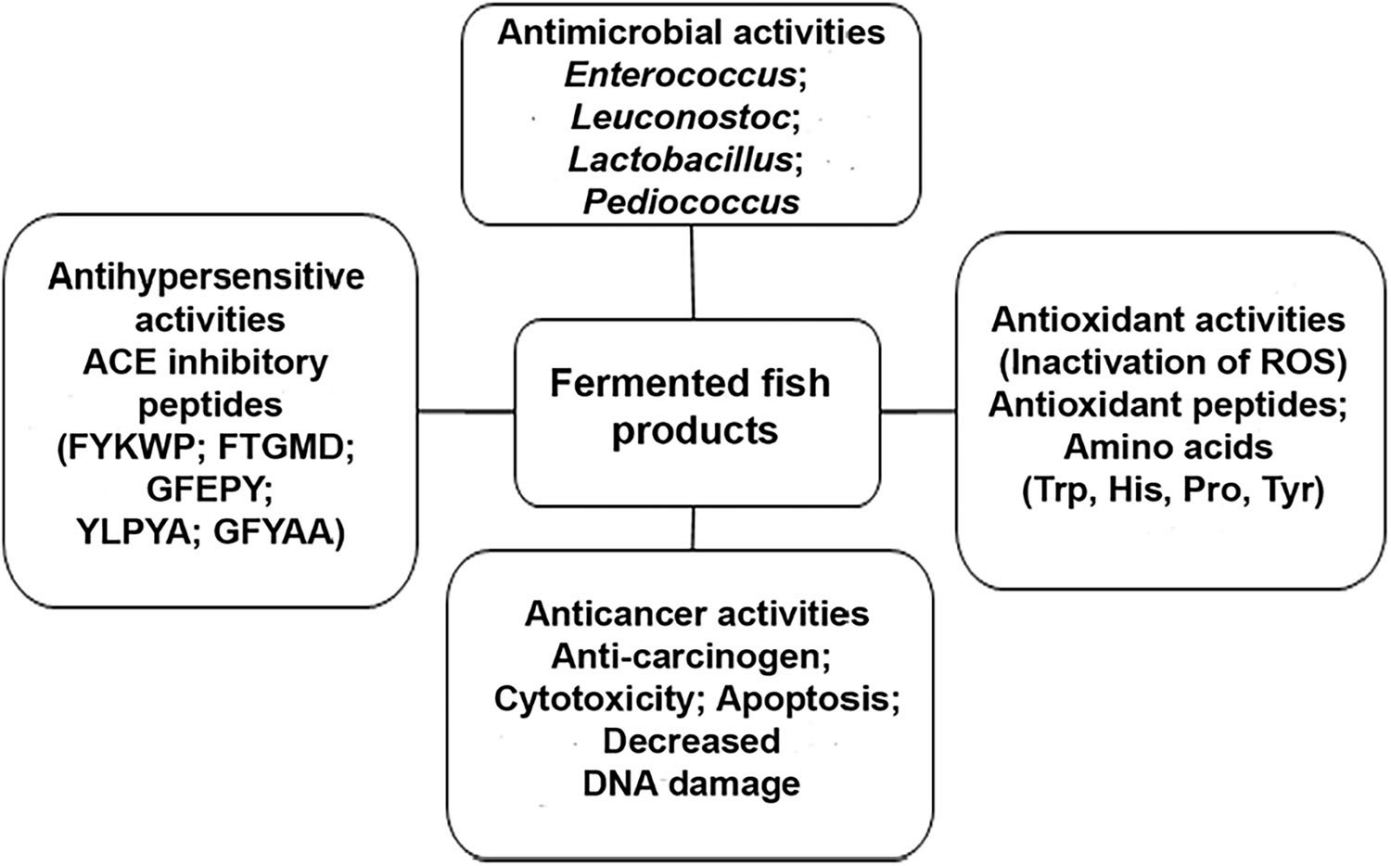
Various health benefits of fermented fish products.

Probiotics and commensal microorganisms synthesize bioactive fatty acids such as conjugated linoleic acid (CLA) and short‐chain fatty acids (SCFAs), which have a benefit on intestinal health (Taylor et al. 2020). LAB also generates antimicrobial metabolites like bacteriocins and peptides, which inhibit harmful bacterial growth (Benítez‐Chao et al. 2021). LAB isolated from ngari, tuntap, and hentak, such as *L. plantarum*, *Lb. fructosus*, *Lb. amylophilus*, *E. faecium*, and *Lc. lactis* subsp. cremoris demonstrated resilience due to their acid and bile tolerance and strong adhesion properties (Abdhul et al. 2014). Probiotics aid gut health by enhancing digestion, reducing discomfort, and mitigating antibiotic‐related diarrhea. Fermented fish products like utonga‐kupsu are reliable sources of probiotics. Strains like *Staphylococcus carnosus*, *S. condimenti*, and *S. piscifermentans* exhibit high tolerance to gastric juice and bile salts (Singh et al. 2018). Aarti et al. (2017) reported that in vitro studies the isolated *Lactobacillus brevis* LAP2 strain, isolated from fermented fish hentak, withstands extreme GI tract acidity and bile salts for extended periods, surviving up to 3 h in stomach acid and 48 h in bile. In addition, the isolated strain exhibited significant antibiotic activity, auto aggregation ranged from 35% to 56% and cell surface hydrophobicity of 35%. The probiotic strain was able to survive under simulated gastric conditions, including low pH (2.0–9.0), exhibited high adhesion rates of 62% and 58%, and showed antagonistic activity against the pathogens Edwardsiella tarda, Streptococcus dysgalactiae, Streptococcus iniae, and Lactococcus garvieae. The study reported that the isolated was identified as Lactococcus lactis and demonstrated promising gut‐related probiotic properties based on in vitro assays (Linh et al. 2018).

### 8.4 Antihypertensive Properties

Globally, hypertension is a leading risk factor for cardiovascular disease and premature mortality, and it is frequently linked to the activity of the ACE. Fermented fish products, which generate numerous BPs during processing, are recognized as a potential source of natural ACE inhibitors for treating hypertension, primarily in vitro offering advantages over synthetic drugs due to fewer side effects (Song et al. 2021). ACE is crucial for regulating blood pressure by modulating the renin–angiotensin (RAS) and kinin–nitric oxide (KNOS) systems, making ACE inhibition a central focus of antihypertensive research. Notably, Thai fermented shrimp pastes contain two dipeptides, Ser‐Val, and Ile‐Phe that inhibit ACE activity with IC_50_ values ranging from 60.68 to 70.03 µM, respectively. This evidence suggested that the BPs could serve as potential sources of antihypertensive and might contribute to the reduction of systolic blood pressure (Kleekayai et al. 2015). In vivo experiments demonstrated that oral administration of a peptide isolated from fermented tuna at a dose of 200 mg/kg BW, decreased protease activity and ameliorated heart tissue damage in hypertensive rats induced by deoxycorticosterone acetate (DOCA) salt (Nurmahdi et al. 2017). In vivo study, oral administration purified peptides at a dose of 100 mg/kg BW dose significantly reduced systolic blood pressure (SBP) by 43 mm Hg in spontaneously hypertensive rats significantly exhibited strong antihypertensive activity. These findings suggested that the purified peptides could be used as a promising source for nutraceuticals and functional foods application (J. Chen et al. 2016). Similarly, the purified peptide Val‐Glu‐Leu‐Tyr‐Pro reduced SBP by 27.6 mm Hg and diastolic blood pressure by 14.7 mm Hg at a 10 mg/kg dose in hypertensive rats (Balti et al. 2015). Administration of salmon gelatin hydrolysate at a dose of 50 mg/kg BW to spontaneously hypertensive rats significantly reduced heart rate, as well as systolic, diastolic, and mean arterial blood pressure in vitro study. Additionally, four purifies peptides (Gly‐Gly‐Pro‐Ala‐Gly‐Pro‐Ala‐Val, Gly‐Pro‐Val‐Ala, Pro‐Pro and Gly‐Phe) and two FAAs (Arg and Tyr) also demonstrated strong ACE inhibition, with IC_50_ values ranged from 0.24 to 1.16 mg/mL and prolyl oligo peptidase (PO) inhibition between 3.30 and 9.57 mg/mL (Neves et al. 2017). In vitro studies protein hydrolysate isolated from horse mackerel and small‐spotted catshark exhibited the highest antihypertensive activity with IC_50_ values of 279 and 302 µg/mL, respectively. Nevertheless, further in vivo studies are required to confirm the ACE inhibitory activity of these peptides (García‐Moreno et al. 2015). Moreover, a peptide composed of the sequence Val‐Ile‐Ser‐Asp‐Glu‐Asp‐Gly‐Val‐Thr‐His, exhibited a higher ACE inhibitory activity (IC_50_ of 8.16 µM), demonstrating its potential application in pharmaceutical and functional food formulations (Chen et al. 2018; Table 4).

**TABLE 4:** Summary of in vitro and in vivo studies, fermentation, and probiotic studies related to fermented fish products.

Model	Dose	Analysis/parameter	Main outcomes	Conclusion	References
In vitro gastrointestinal digestion	0.5 g / 40 mL digestible volume	INFOGEST 2.0 protocol, HPLC, FESEM, UV visible spectrophotometer, SFM	↑Bacteroidetes ↓Firmicutes, ↓Parabacteroides, ↓Prevotella‐9 ↑DPPH ↑ABTS	In vitro fermentation using *M. purpureus* on fish protein enriched with free amino acids significantly altered the fermentation metabolites profiles and gut microbial composition while also enhancing the digestion rate of fish protein under gastric conditions.	Liu et al. (2022)
In vitro rumen fermentation	LAB: 1.02×10^1^ ^1^ CFU/mL; Yeast: 1.5×10^10^ CFU/mL	pH meter, gravimetric method, colorimetric assay, gas chromatography	↑DMD (63%–70%) ↑ OMD (64%–71%) ↑VFA 86–121 mM, ↑NH_3_ (12.3–16.8 mM) ↑pH ∼6.86–7.12	The LAB S. harbinensis LH991 and the yeast P. kudriavzevii B‐5P demonstrated strong probiotic candidates in vitro rumen fermentation.	Ardani et al. (2023)
In vitro fermentation	1 mL bacterial inoculum in 40 mL broth	pH, Gram staining, catalase test, API ZYM enzymatic assay, PCR (16S rRNA), antifungal assays, MIC, MFC determination	↑LAB growth (up to 13 log CFU/mL) ↓pH (from 6.9 to 3.7) Strong antifungal activity (MIC: 1–16 g/L; MFC: 8–32 g/L) Highest for L. plantarum strains (S3, S4)	Processing of sea bass meat and by‐products through fermentation with proteolytic *L. plantarum* enhanced the production of antifungal metabolites, which can act as natural food preservatives.	Martí‑Quijal et al. (2020)
In vitro gastrointestinal simulation	1% (10^7^–10^8^ CFU/g) inoculum of Enterococcus spp.	Spectrophotometric method, pH meter, thiobarbituric acid assay, MTT assay	↑ LAB count ↓ pH ↓ TBARs ↑ Proteolysis ↑ Antioxidant activity ↑ α‐Amylase inhibition ↑ α‐Glucosidase inhibition ↑ Cytotoxicity (Caco‐2, MCF‐7) ↑ Cholesterol removal ↑ Bile salt hydrolysis ↑ EPS production	Fermentation with *Enterococcus* spp. enhanced proteolysis and produced bioactive peptides with antioxidant, antidiabetic, and cytotoxic potential, indicating probiotic value in functional meat products	AlKalbani et al. (2019)
In vitro keratinocytes and melanocyte coculture. In vivo (HRM‐2 mice	250 µg/mL FC in cell model; 150, 250, 350 mg/kg FC in animal model	Western blot, ELISA, colorimetry, Fontana–Masson strain	In vitro ↑ GlyR and GlyT ↓ NOX (1/2/4) ↑ GSH/GSSG ratio ↑ SOD ↓ 8‐OHdG ↓ p‐p38/p38, PKC, MITF, TRP‐1, TRP‐2, TYR In vivo ↑ GlyR and GlyT ↓ NOX1/2/4 ↑ GSH/GSSG ratio ↑ SOD activity ↓ pp38/p38, ↓ PKC, ↓ MITF, ↓ TRP‐1, ↓ TRP‐2, ↓ TYR ↓ Melanin content ↑ Skin lightness	Fermented fish collagen attenuates UV‐induced melanogenesis by reducing oxidative stress through glycine‐mediated upregulation of GlyR/GlyT and downregulation of melanogenesis pathways	Byun et al. (2024)
In vitro cell line assays	10^6^–10^9^ CFU/mL of L. plantarum MKTJ24	pH, GC–MS, spectrophotometer, RT‐PCR, DCFH‐DA fluorescence	↑ Survival rate under gastric and bile salt simulation ↑ Antagonistic activity ↑ Self‐aggregation and hydrophobicity ↑ Adhesion to HT‐29 cells ↑ Mucin gene expression ↓ NO and ↓ ROS ↑ DPPH	L. plantarum MKTJ24 is a promising probiotic candidate with anti‐inflammatory and immunomodulatory properties relevant for fermented fish product valorization	Joishy et al. (2024)
In vitro digestion model	Starter culture inoculation 1% (v/w); ∼7–8 log CFU/mL of each strain; 5 g fermented fish per digestion run	HPLC‐DAD, GC–MS/MS, RP‐HPLC	↓ LAB, yeast during gastric phase ↑ LAB, yeast in the small intestine ↑/↓ Biogenic amines PUT, CAD, TYR ↑ during the small intestine ↓ In inoculated groups ↓ Nitrite in the gastric digestion ↑ Nitrite during small intestine ↑ NDMA, NPIP ↓ NDMA	Coculturing with *S. xylosus* 135, L. plantarum 120, and S. cerevisiae 2018 reduced N‐nitrosamine formation and biogenic amine levels during in vitro digestion, thereby improving the safety of fermented fish.	Li et al. (2024b)
In vitro keratinocytes In vivo HRM‐2 mice	250 µg/mL in cells; 150, 250, 350 mg/kg in mice	ELISA, immunostaining, western blot, Masson trichrome, BAB staining	In vitro ↓ AGE–RAGE receptors ↓ TNF‐α ↓ NF‐κB nuclear translocation ↑ Cell viability ↓ Cytotoxicity In vivo ↓ AGE–RAGE receptors ↓ TNF‐α ↓ NF‐κB nuclear translocation ↓ MMP (1, 3, 9) ↓ Smad7 ↑ Collagen (I, III) ↑ Collagen fiber accumulation ↑ Skin moisture and elasticity	Fermented fish collagen mitigates photoaging by attenuating AGE–RAGE binding and its downstream pathways, thereby exhibiting potent antiphotoaging efficacy	Oh et al. (2024)
In vitro digestion of cooked fermented fish	—	AOAC (2003), GC–MS, spectrophotometer, SDS‐PAGE profiling	↑ Protein ↓ Moisture content ↑ Fat ↑ PUFA ↑ Degree of hydrolysis after digestion ↓ DPPH scavenging activity ↓ Metal chelating activity after digestion ↑ Reducing power	Different cooking methods altered antioxidant behavior but did not impair digestibility or bioactive potential	Hanjabam et al. (2020)
In vitro cytotoxicity assays	LAB isolates from fermented fish; 10^6^–10^8^ CFU/mL	pH, spectrophotometer, microscopic observation, MTT assay, DAPI staining, flow cytometry, DCFH‐DA fluorescence assay	↑ Survival in simulated gastric ↑ Self‐aggregation and cell surface hydrophobicity ↑ Adhesion to HT‐29 and Caco‐2 intestinal cell lines ↑ Antimicrobial activity ↓ HT‐29 and Caco‐2 cancer cell ↑ Apoptosis markers ↓ ROS accumulation ↑ Antioxidant activity	Probiotic isolated from traditional fermented fish exhibited strong antimicrobial effects. L. plantarum and L. rhamnosus show the highest cytotoxicity against colon cancer cells through apoptosis induction, and oxidative stress reduction.	Singh et al. (2018)
In vitro antagonistic assay	L. pentosus strain LAP1 selected; inoculum 1% in optimized media	Agar well diffusion, pH, temperature, carbon and nitrogen source, supplements, spectrophotometer	↑ Antibacterial activity ↑ Activity at pH 5 ↑ Production with lactose as carbon source and ammonium chloride as nitrogen source ↓ Activity at alkaline pH (> 7) ↑ DPPH scavenging	L. pentosus LAP1 produced proteinaceous, heat‐sensitive antibacterial substances against S. epidermidis, M. luteus, S. flexneri, Y. enterocolitica, and P. vulgaris with antioxidant potential, and useful for food preservation	Aarti et al. (2016)
In vitro	2 mg/mL (for antibacterial assay) and 200–400 µg/mL (for anticancer assay).	Gel filtration chromatography, UV spectrophotometry, MTT assay,	↑ Protein concentration in fraction 3 ↓ Conductivity in fractions 2–4 ↑ Antibacterial activity ↓ Bacterial growth (especially S. aureus and E. coli) ↓ Antibacterial effect after Proteinase K digestion ↓ Cytotoxicity on HepG2	Protein hydrolysates isolated from fish sauce byproducts contained bioactive peptides with inhibitory effects against, *E*. *coli*, *X*. *oryzae* pv. *Oryzae and S*. *aureus* but showed no cytotoxicity impact toward HepG2 or Vero cell lines	Khositanon et al. (2018)
In vitro assay	CFS from W. confusa at IC_50_ = 27 ± 1.5 µg/m	qRT‐PCR, Gram staining, MTT assay (HT‐29 cells), flow cytometry, UV‐Vis spectrophotometer, fluorescence microscopy	↑ Survival at low pH (2–3) ↑ bile salt tolerance (0.3%–1%) ↑ Autoaggregation ↑ cell surface hydrophobicity ↓ Hemolytic activity ↑ Antimicrobial activity ↑ DPPH and ABTS scavenging activity ↓ Viability of HT‐29 cells ↑ Apoptosis ↓ Bcl‐2 ↑ BAX	The cell‐free supernatant (CFS) of *W. confusa* exhibited potent inhibitory potential against *C*. *albicans*, *S*. *aureus*, *K*. *pneumoniae* along with strong antioxidant and anticancer effects through apoptosis induction	Sreelakshmi et al. (2025)
In vitro study	—	Gram staining, catalase test, PCR amplification, BLAST analysis, UV–Vis spectrophotometer, pH meter, centrifuge	↑ Survival at low pH (2–3) ↑ bile tolerance (0.3%–1%) ↑ Cell surface hydrophobicity (27%–41%) ↑ auto‐aggregation ↓ Hemolytic activity ↑ Antimicrobial activity ↑ DPPH radical scavenging activity ↓ Antibiotic resistance	The LAB isolated from shidal exhibited potential probiotic attributes, including survival under gastrointestinal‐like conditions, antimicrobial activity, and aggregation. *L. plantarum* and *P*. *pentosaceus* were identified as the most promising for use as probiotic cultures in functional fermented fish products	Gupta et al. (2021)
In vitro antimicrobial assay	—	Gel electrophoresis and BLAST analysis, catalase, PCR amplification, 16S rRNA sequencing, well diffusion assay, bacteriocin neutralization assay, light microscopy	↑ Antimicrobial activity against E. coli O157:H7 (21.26 mm) ↑ S. aureus ATCC 25923 (18.23 mm) ↓ Listeria monocytogenes (5.10 mm) ↑ Crude bacteriocin activity ↓ No activity against L. monocytogenes ↓ pH (acidic medium)	Pediococcus acidilactici PB22 isolated from bekasam exhibited strong antimicrobial activity, primarily due to bacteriocin and organic acid production.	Purwati et al. (2019)
In vitro antagonistic assay	20 µL LAB culture and 50 µL neutralized cell‐free supernatant	Incubator (37°C), centrifuge, pH meter, spectrophotometer, thermal cycler, and electrophoresis unit	↑ Isolates showed inhibition against S. aureus ATCC 6538 and P. aeruginosa ATCC 27853 ↑ Strongest inhibition by the isolate Aerococcus NJ‐20 ↓ No inhibition against P. aeruginosa ↓ No bacteriocin activity ↑ NJ‐20 tolerant to 6.5% NaCl and pH 9.6 ↓ Growth at pH 4.4 or 18% NaCl	LAB isolates from peda exhibited varying levels of antagonistic activity, with *Aerococcus* NJ‐20 showing the strongest inhibition against *S. aureus*.	Putra et al. (2018)
In vitro	—	AOAC methods, spectrophotometer, SDS–PAGE, LC‐MS/MS, scanning electron microscopy (SEM)	↑ DH% after L. casei fermentation ↑ DPPH and ABTS scavenging activity ↑ FRAP and metal ion chelation ↑ Antibacterial activity ↓ Lipid oxidation (TBARS) ↑ Small peptide generation ↓ Pathogenic bacterial growth	Fermentation of fish sauce by indigenous *L*. *casei* enhanced radical scavenging and inhibitory activities due to the production of bioactive peptides	Abd Rashid et al. (2022)
In vitro screening of LAB from fermented fish	—	UV–Vis spectrophotometer, amino acid analyzer, HPLC, gas chromatograph (GC‐FID), PCR thermal cycler	↑ Amino acid release ↓ Ammonia and indole levels during digestion ↑ Propionic acid in fermentation ↑ Parabacteroides abundance during colon fermentation	Application of *Monascus* improved in vitro digestibility and modulated fermentation metabolites and microbiota, reducing harmful nitrogenous compounds	Amarantini et al. (2019)

## 9 Food Safety Hazard in Fermented Fish Products

A significant food safety issue associated with fermented fish products is the generation of biogenic amines, specifically histamine, cadaverine, putrescine, and tyramine. These substances primarily originate from microbial decarboxylation of amino acids during fermentation and subsequent storage, particularly when temperature control is inadequate, salting is insufficient, or fermentation duration is prolonged (Ashaolu et al. 2023). Elevated concentrations of biogenic amines can precipitate detrimental health consequences, encompassing headaches, hypertension, allergic responses, and histamine poisoning, especially among susceptible individuals (Omer et al. 2021). The concentration of these amines is closely linked to microbial composition, salt concentration, and the hygienic practices employed during processing (Anyogu et al. 2021; Ayeloja and Jimoh 2022). When hygienic practices are deficient, pathogenic and spoilage microorganisms pose additional threats to the safety of fermented fish. While fermentation and salting can mitigate many pathogens, they do not eliminate all microbial hazards. Reports have documented contamination with *Enterobacteriaceae*, *S. aureus*, *Salmonella* spp., *E. coli, and* other opportunistic pathogens in various African fermented fish products. These microorganisms may be introduced via contaminated raw materials, unsafe water sources, unsanitary drying surfaces, or inadequate storage conditions. Furthermore, in open‐air fermentation and sun‐drying processes, exposure to dust, insects, and animals exacerbates the risk of microbial contamination, thereby raising public health concerns (Osman et al. 2022).

Chemical contamination, particularly heavy metals such as mercury, lead, cadmium, and arsenic, is another crucial concern that often receives insufficient attention. Fish sourced from contaminated coastal areas or inland water bodies are susceptible to bioaccumulation of these metals, which remain stable during fermentation and subsequent processing (Samarajeewa 2023; Sonone et al. 2020). Unlike microbial threats, heavy metals cannot be eliminated by salting, fermentation, or drying methods (Millena et al. 2023). Consequently, chronic exposure resulting from the consistent consumption of fermented fish may present enduring health hazards, encompassing neurotoxicity, renal impairment, and potential carcinogenic consequences. Furthermore, parasitic contamination, including nematodes, has been observed in inadequately processed products, particularly when salting and drying procedures fail to effectively inactivate larvae (Abera and Adimas 2024). Notwithstanding these potential hazards, fermented fish retains its nutritional significance, as it is abundant in high‐quality proteins, essential fatty acids, and minerals. Furthermore, numerous products demonstrate functional attributes, encompassing antimicrobial, antioxidant, antihypertensive, and flavor‐enhancing properties. Consequently, the enforcement of health regulations, the enhancement of hygiene during preparation, display, and storage, and the promotion of sound manufacturing practices among small‐scale producers are of paramount importance (Anyogu et al. 2021; Ayeloja and Jimoh 2022). Fish smoking and fermentation are economically significant in the region, contributing to employment and minimizing postharvest waste. Nevertheless, the sector faces challenges, including higher fuel costs, outdated smoking methods, and limited access to advanced processing infrastructure (Bi et al. 2022; Gutema and Hailemichael 2021). These limitations often lead to substandard processing practices, thereby amplifying rather than alleviating safety hazards. Globally, fermented foods and beverages are valued for their high protein, vitamin, and mineral content, and sustainable, ethical production methods are crucial, especially in resource‐limited settings (Khalfallah et al. 2023).

## 10 Future Prospects and Challenges

Fermented fish products have gained increasing research attention due to their nutritional value, functional properties, and cultural importance. However, scientific evidence supporting their health benefits remains limited compared with other fermented foods. Future research should focus on validating the biological activities of fermentation‐derived bioactive compounds, particularly peptides, through well‐designed in vivo studies and, where feasible, human investigations. Establishing their stability, bioavailability, and dose‐dependent effects is essential to support health claims and enable their application in functional food development.

A major challenge in fermented fish production is the variability associated with traditional, spontaneous fermentation processes. While these methods contribute to distinctive sensory characteristics, they often result in inconsistent product quality and safety. The use of well‐defined starter cultures, including selected strains of LABs, *Bacillus* spp., and *Monascus purpureus*, offers a promising approach to improve process control. Future studies should aim to optimize fermentation conditions and tailor starter cultures to specific fish substrates in order to enhance BP generation, improve digestibility, and reduce the formation of undesirable metabolites. Advances in omics‐based technologies, such as metagenomics, peptidomics, and metabolomics, provide valuable tools for understanding microbial dynamics and biochemical transformations during fermentation. Integrating these approaches can help identify key microorganisms and metabolites responsible for functional properties and flavor development, while also supporting predictive strategies for quality and safety assessment. Food safety remains a critical concern, particularly regarding biogenic amines, pathogenic microorganisms, and hygiene‐related contamination. Strengthening sanitation practices, standardizing processing protocols, and implementing robust monitoring systems are essential for ensuring consumer safety. Therefore, interdisciplinary research integrating fermentation science, nutrition, food safety, and sustainability will be vital for advancing fermented fish products toward broader acceptance and industrial application.

## 11 Conclusion

Fermented fish processes are gaining research interest due to their diverse production methods and beneficial biological activities. These products possess significant potential for development of innovative fish products with enhanced sensory attributes, nutritional composition, and various therapeutic properties. These products are essential dietary staples, crucial for local economies and cultural heritage, embodying innovative food preservation techniques passed down through generations. Key development areas include optimizing fermentation conditions, evaluating nutritional content, and improving safety with advanced practices and technology. Advances in omics and processing technology will be essential for market growth and sustainability. Despite limited scientific research compared to other fermented foods, they offer bioactive compounds that enrich nutrition and culinary experiences. Preserving these traditions promotes sustainable food practices and preserves cultural diversity on a global scale. Fermented fish products contain numerous bioactive compounds, anticancer, and antimicrobial peptides. They may act as natural ACE inhibitors, promoting heart health. LAB produces nonvolatile acids for sourness and preservation, while generating aromatic ketones and aldehydes from lipid and protein breakdown, contributing to diverse flavors like umami. Variations in ingredients and fermentation methods influence these distinct flavors, showcasing LAB's pivotal role in global culinary diversity. Research into fermented fish is critical for both the food industry and public health. It aims to enhance production processes, improve nutritional value, and ensure safety. Implementing modern technologies and rigorous safety measures can mitigate risks associated with fermentation, thus maintaining high‐quality standards and product safety for consumers worldwide.

## Author Contributions

**Banlambhabok Khongthaw**: conceptualization, writing – review and editing, writing – original draft, methodology, supervision, data curation, formal analysis, validation, investigation. **Mthokozisi Dladla**: writing – review and editing, writing – original draft, investigation, data curation, formal analysis. **Pankaj Kumar** **Chauhan**: writing – review and editing, writing – original draft, data curation, formal analysis, investigation. **Kanika Dulta**: writing – review and editing, writing – original draft, investigation, data curation, formal analysis. **Vinod Kumar**: data curation, formal analysis, writing – review and editing, writing – original draft, investigation. **Helen Oneyaka**: supervision, investigation, writing – original draft, writing – review and editing, formal analysis, data curation. **Soumya Ghosh**: supervision, validation, methodology, formal analysis, data curation, writing – review and editing, writing – original draft.

## Funding

The authors have nothing to report.

## Ethics Statement

Ethics approval was not required for this research.

## Conflicts of Interest

The authors declare no conflicts of interest.

## Data Availability

Data will be made available upon request.
